# Healthy lifestyle and life expectancy free of major chronic diseases at age 40 in China

**DOI:** 10.1038/s41562-023-01624-7

**Published:** 2023-07-10

**Authors:** The China Kadoorie Biobank Collaborative Group, Qiufen Sun, Yizhen Hu, Canqing Yu, Yu Guo, Pei Pei, Ling Yang, Yiping Chen, Huaidong Du, Dianjianyi Sun, Yuanjie Pang, Sushila Burgess, Sam Sansome, Feng Ning, Junshi Chen, Zhengming Chen, Liming Li, Jun Lv, Members of the China Kadoorie Biobank collaborative group, International Steering Committee:, Junshi Chen, Zhengming Chen PI, Robert Clarke, Rory Collins, Yu Guo, Liming Li PI, Jun Lv, Richard Peto, Robin Walters, International Co-ordinating Centre, Oxford:, Daniel Avery, Derrick Bennett, Ruth Boxall, Sue Burgess, Ka Hung Chan, Yumei Chang, Yiping Chen, Zhengming Chen, Johnathan Clarke, Robert Clarke, Huaidong Du, Ahmed Edris Mohamed, Zammy Fairhurst-Hunter, Hannah Fry, Simon Gilbert, Alex Hacker, Mike Hill, Michael Holmes, Pek Kei Im, Andri Iona, Maria Kakkoura, Christiana Kartsonaki, Rene Kerosi, Kuang Lin, Mohsen Mazidi, Iona Millwood, Sam Morris, Qunhua Nie, Alfred Pozarickij, Paul Ryder, Saredo Said, Sam Sansome, Dan Schmidt, Paul Sherliker, Rajani Sohoni, Becky Stevens, Iain Turnbull, Robin Walters, Lin Wang, Neil Wright, Ling Yang, Xiaoming Yang, Pang Yao, National Co-ordinating Centre, Beijing:, Yu Guo, Xiao Han, Can Hou, Jun Lv, Pei Pei, Chao Liu, Canqing Yu, Qingmei Xia, 10 Regional Co-ordinating Centres: Qingdao CDC:, Zengchang Pang, Ruqin Gao, Shanpeng Li, Haiping Duan, Shaojie Wang, Yongmei Liu, Ranran Du, Yajing Zang, Liang Cheng, Xiaocao Tian, Hua Zhang, Yaoming Zhai, Feng Ning, Xiaohui Sun, Feifei Li, Licang CDC:, Silu Lv, Junzheng Wang, Wei Hou, Heilongjiang Provincial CDC:, Wei Sun, Shichun Yan, Xiaoming Cui, Nangang CDC:, Chi Wang, Zhenyuan Wu, Yanjie Li, Quan Kang, Hainan Provincial CDC:, Huiming Luo, Tingting Ou, Meilan CDC:, Xiangyang Zheng, Zhendong Guo, Shukuan Wu, Yilei Li, Huimei Li, Jiangsu Provincial CDC:, Ming Wu, Yonglin Zhou, Jinyi Zhou, Ran Tao, Jie Yang, Jian Su, Suzhou CDC:, Fang Liu, Jun Zhang, Yihe Hu, Yan Lu, Liangcai Ma, Aiyu Tang, Shuo Zhang, Jianrong Jin, Jingchao Liu, Guangxi Provincial CDC:, Mei Lin, Zhenzhen Lu, Liuzhou CDC:, Lifang Zhou, Changping Xie, Jian Lan, Tingping Zhu, Yun Liu, Liuping Wei, Liyuan Zhou, Ningyu Chen, Yulu Qin, Sisi Wang, Sichuan Provincial CDC:, Xianping Wu, Ningmei Zhang, Xiaofang Chen, Xiaoyu Chang, Pengzhou CDC:, Mingqiang Yuan, Xia Wu, Xiaofang Chen, Wei Jiang, Jiaqiu Liu, Qiang Sun, Gansu Provincial CDC:, Faqing Chen, Xiaolan Ren, Caixia Dong, Maiji CDC:, Hui Zhang, Enke Mao, Xiaoping Wang, Tao Wang, Xi zhang, Henan Provincial CDC:, Kai Kang, Shixian Feng, Huizi Tian, Lei Fan, Huixian CDC:, XiaoLin Li, Huarong Sun, Pan He, Xukui Zhang, Zhejiang Provincial CDC:, Min Yu, Ruying Hu, Hao Wang, Tongxiang CDC:, Xiaoyi Zhang, Yuan Cao, Kaixu Xie, Lingli Chen, Dun Shen, Hunan Provincial CDC:, Xiaojun Li, Donghui Jin, Li Yin, Huilin Liu, Zhongxi Fu, Liuyang CDC:, Xin Xu, Hao Zhang, Jianwei Chen, Yuan Peng, Libo Zhang, Chan Qu

**Affiliations:** 1 Department of Epidemiology & Biostatistics, School of Public Health, Peking University, Beijing 100191, China; 2 Peking University Center for Public Health and Epidemic Preparedness & Response, Beijing 100191, China; 3 Key Laboratory of Epidemiology of Major Diseases (Peking University), Ministry of Education, Beijing 100191, China; 4 Fuwai Hospital Chinese Academy of Medical Sciences, Beijing, China; 5 Medical Research Council Population Health Research Unit at the University of Oxford, Oxford, United Kingdom; 6 Clinical Trial Service Unit & Epidemiological Studies Unit (CTSU), Nuffield Department of Population Health, University of Oxford, United Kingdom; 7 NCDs Prevention and Control Department, Qingdao CDC, Qingdao, Shandong, China; 8 China National Center for Food Safety Risk Assessment, Beijing, China

## Abstract

Whether a healthy lifestyle helps achieve gains in life expectancy (LE) free of major non-communicable diseases and its share of total LE in Chinese adults remains unknown. We considered five low-risk lifestyle factors: never smoking or quitting for reasons other than illness, no excessive alcohol use, being physically active, healthy eating habits, and healthy body fat levels. Here, we show that after a median follow-up of 11.1 years for 451,233 Chinese adults, the LE free of cardiovascular diseases, cancer, and chronic respiratory diseases (95% confidence interval) at age 40 for individuals with all five low-risk factors was on average 6.3 (5.1-7.5) years (men) and 4.2 (3.6-5.4) years (women) longer than those with 0-1 low-risk factors. Correspondingly, the proportion of disease-free LE to total LE increased from 73.1% to 76.3% for men and from 67.6% to 68.4% for women. Our findings suggest that promoting healthy lifestyles could be associated with gains in disease-free LE in the Chinese population.

## Introduction

Life expectancy (LE) in China has risen in the past 30 years.^1^ The aging population and the widespread prevalence of health risk factors drive a rise in the burden of non-communicable diseases (NCDs). Meanwhile, advances in medicine play an important role in reducing mortality from NCDs. As a result, more and more people live with chronic conditions, such as cardiovascular diseases (CVDs), cancer, and chronic respiratory diseases (CRDs).^2^ Healthy life expectancy (HLE) adds a quality-of-life dimension to the estimates of LE by dividing the expected lifespan into life years spent with and without diseases. According to the compression of morbidity hypothesis, healthy aging should manifest as a greater extension of LE free of diseases than total LE or an increase in its share of total LE.^3^ Therefore, LE free of diseases can be a better indicator than total LE to monitor progress in healthy aging and the achievement of sustainable development goals.^4^

A healthy lifestyle has been associated with lower incidence risks of CVDs, cancer, and CRDs and mortality risks from these diseases,^5–7^ thus being associated with a delayed onset of diseases and longer LE.^8^ There is an increasing interest in understanding the net effect of a healthy lifestyle on the proportion of disease-free LE to the total LE. Available studies were mainly conducted in Western countries. Most studies focused on individual lifestyle factors or LE free of a certain ill-health state such as CVDs,^9–11^ or type 2 diabetes (T2D).^12^ Only three studies assessed the impact of combined lifestyle factors on LE free of several NCDs.^8,13,14^ In the context of different economic and social development and determinants of health, it remains unclear how increasing the adoption of a healthy lifestyle through public health interventions helps achieve gains in LE free of major chronic diseases and healthy aging in the Chinese population.

The present study examined the impact of individual and combined lifestyle factors on the LE free of major NCDs and its share of total LE in the China Kadoorie Biobank (CKB) of 0.5 million Chinese adults. The NCDs of interest are CVDs, cancer, and CRDs (including chronic obstructive pulmonary disease [COPD] and asthma), which have posed a heavy burden on healthcare systems in China.^15^

## Results

### Characteristics of the study population

A total of 451,233 participants were included in this study, with a mean baseline age of 51.0 ± 10.4 years and 40.2% being males. The proportion of participants adopting at least three, four, and all five low-risk lifestyle factors were 68.6%, 28.2%, and 2.0%, respectively. Overall, younger women and better-educated participants were more likely to adopt low-risk lifestyles (Supplementary Table 1). Among 22,275 participants who participated in the 2013-14 resurvey, the risk level of lifestyle factors remained largely unchanged over the mean follow-up period of 8 years, regardless of disease occurrence in this period (Supplementary Table 2).

During a median follow-up of 11.1 years (4.93 million person-years), we documented 111,002 new CVD cases, 24,635 cancer cases, and 12,506 CRD cases. When considering CVDs, cancer, and CRDs as a whole, the number of cases with CVDs as the first disease was 106,065. The corresponding numbers of cases for cancer and CRDs were 20,223 and 8,760, respectively (1,461 participants developed multiple concurrent diseases as the first disease). We documented 34,740 deaths; 4,710 deaths did not have CVDs, cancer, or CRDs being recorded between baseline and death.

### Lifestyle and transitions between states

**Figure 1:**
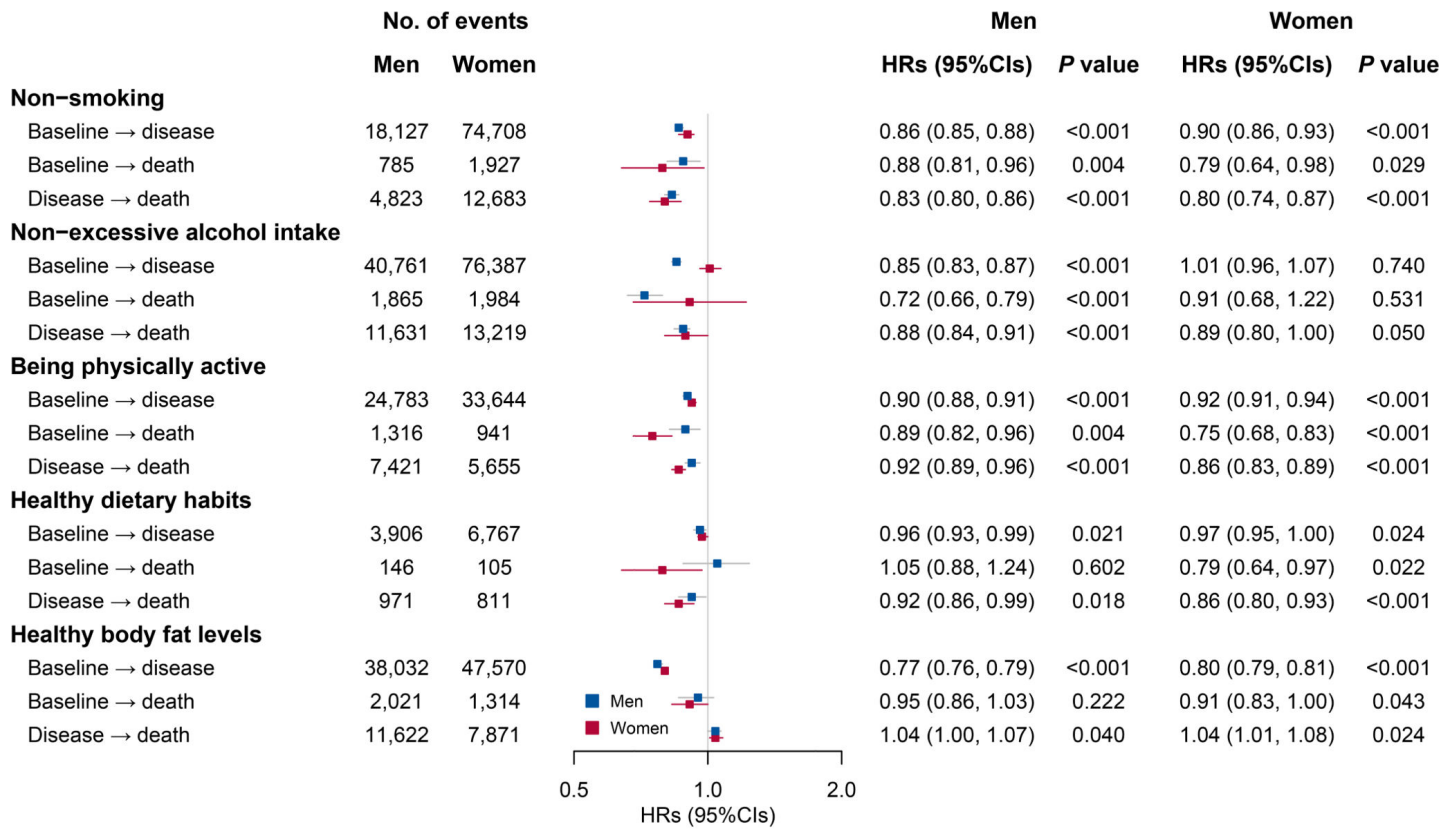
Multivariable-adjusted hazard ratios (95% CIs) for each transition by individual low-risk lifestyle factors in men (n=181,544) and women (n=269,689) separately. HR indicates hazard ratio; CI, confidence interval. Cox regression was used to estimate the HRs and 95% CIs with all five lifestyle factors included simultaneously in the same model, and adjustment for education, marital status, family histories of heart attack, stroke, and cancer, and menopausal status (women only) as appropriate. In the analysis of the transition from a disease state to all-cause mortality, HRs were further adjusted for age at diagnosis of corresponding diseases (years). All statistical tests were two-sided. The definition of low-risk lifestyle factors was the same as in Table 1. The disease event in this analysis refers to the first occurrence of any cardiovascular diseases, cancer, and chronic respiratory diseases (including chronic obstructive pulmonary disease and asthma). The number of events refers to the number of cases in each transition with the corresponding exposure. The effect estimates represent the HRs from the Cox regression and the error bars represent 95% CIs.

In the multivariable-adjusted models, almost all five low-risk lifestyle factors were associated with reduced risks of developing any of the three NCDs in both men and women (Figure 1). Healthy body fat levels, defined by body mass index (BMI) and waist circumference (WC), showed the strongest negative association, with hazard ratios (HRs) and 95% confidence intervals (CIs) of 0.77 (0.76-0.79; *P* <0.001) and 0.80 (0.79-0.81; *P* <0.001) for men and women, respectively. All four low-risk factors other than healthy body fat levels were associated with lower mortality risks for either transition from baseline or the presence of any of the three NCDs.

When low-risk lifestyle factors were considered jointly, there were linear decreasing trends in the incidence and mortality risk with the increasing number of low-risk lifestyle factors for all three transitions (Table 1). The corresponding HRs (95% CIs) per one-factor increase for men were 0.85 (0.84-0.86; *P* <0.001), 0.86 (0.83-0.90; *P* <0.001), and 0.90 (0.89-0.92; *P* <0.001) for baseline to disease, baseline to death, and disease to death, respectively; the corresponding values for women were 0.88 (0.87-0.89; *P* <0.001), 0.83 (0.78-0.88; *P* <0.001), and 0.93 (0.91-0.95; *P* <0.001). The gradient association persisted for analyses of disease-specific outcomes. Sensitivity analyses did not alter the results substantially (Supplementary Table 3).

### Lifestyle and LE with and without chronic diseases

**Figure 2:**
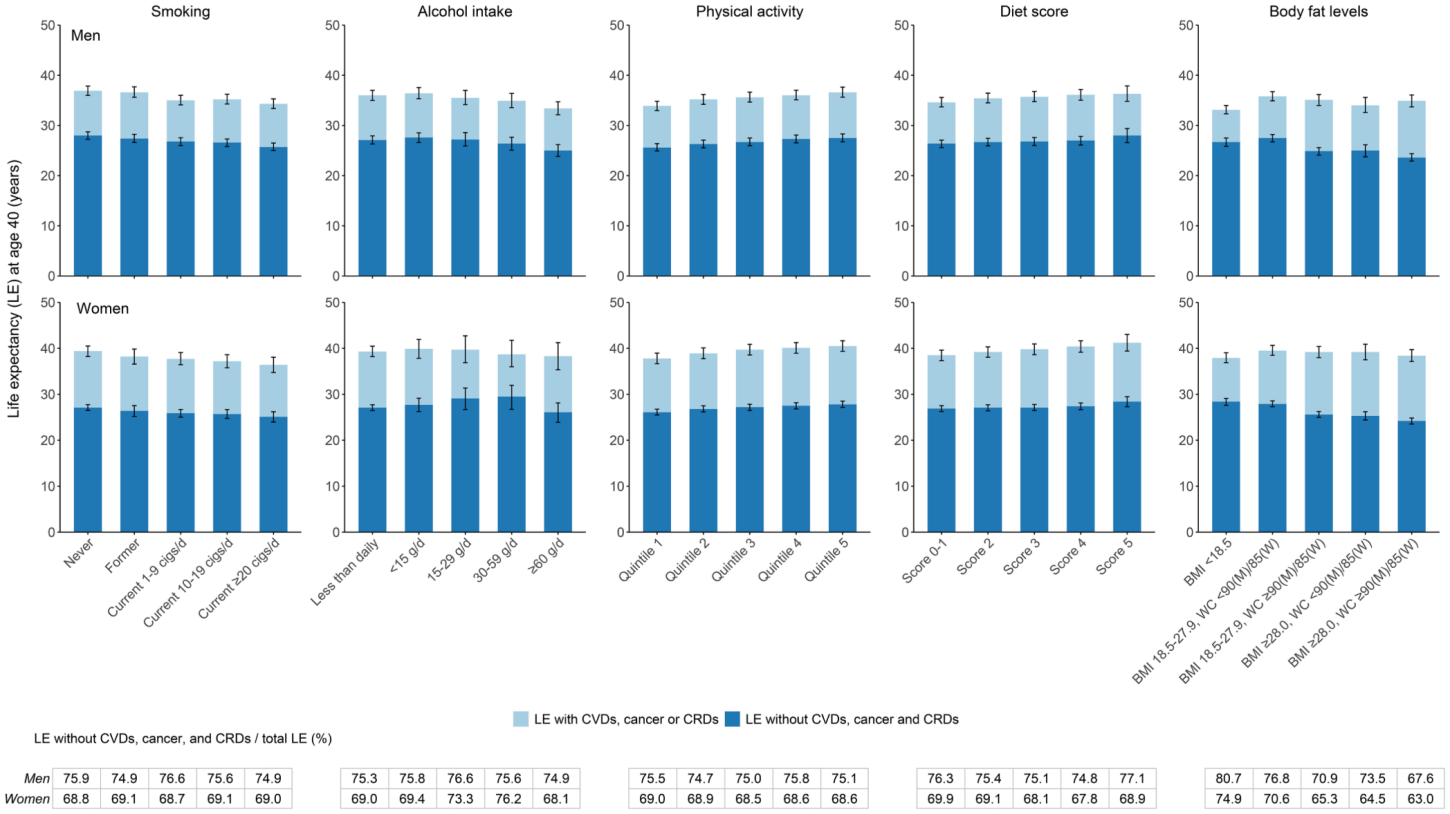
Life expectancy at age 40 years with and without cardiovascular diseases (CVDs), cancer, and/or chronic respiratory diseases (CRDs) by levels of individual lifestyle risk factors in men (n=181,544) and women (n=269,689) separately. Cigs indicate cigarettes or equivalent; BMI, body mass index; WC, waist circumference; M, men; W, women. Former smokers refer to those having stopped smoking for reasons other than illness. Participants who had stopped smoking due to illness were classified as current smokers. Less than daily group included never-regular drinkers and current weekly drinkers. Former alcohol drinkers were included in the heavy drinking category (≥60 g of pure alcohol per day). Physical activity level was categorized based on age- (<50 years, 50-59 years, and ≥60 years) and sex-specific quintile of total physical activity level. Diet score was created based on the following criteria: eating fresh vegetables daily, eating fresh fruits daily, eating red meat 1-6 days per week, eating legumes ≥4 days per week, and eating fish ≥1 day per week. For each food group, the participant who met the criterion received a score of 1, and otherwise, 0. The error bars represent 95% CIs.

The Poisson regression model fitted the observed age-specific rates well for each transition in men and women (Supplementary Figure 1). About three-quarters of the total LE at age 40 in men was free of the three NCDs, but the proportion was lower for women (Supplementary Figure 2). All four low-risk lifestyle factors other than healthy body fat levels were accompanied by a parallel extension of total LE at age 40 and LE free of the three NCDs (Figure 2). Although the most obese participants had about the same LE as those with a BMI of 18.5-27.9 kg/m^2^ and without abdominal obesity, the LE free of the three NCDs of the former was shorter by 3.9 (3.5-4.1) years for men and 3.7 (3.5-4.0) years for women (Supplementary Figure 3). The proportion of LE free of the three NCDs to the total LE was also obviously lower for the most obese participants (men: 67.6%, women: 63.0%), indicating expansion of morbidity (Figure 2). Results of LE with and without each NCD were presented in Supplementary Figures 4, 5, and 6.

**Figure 3:**
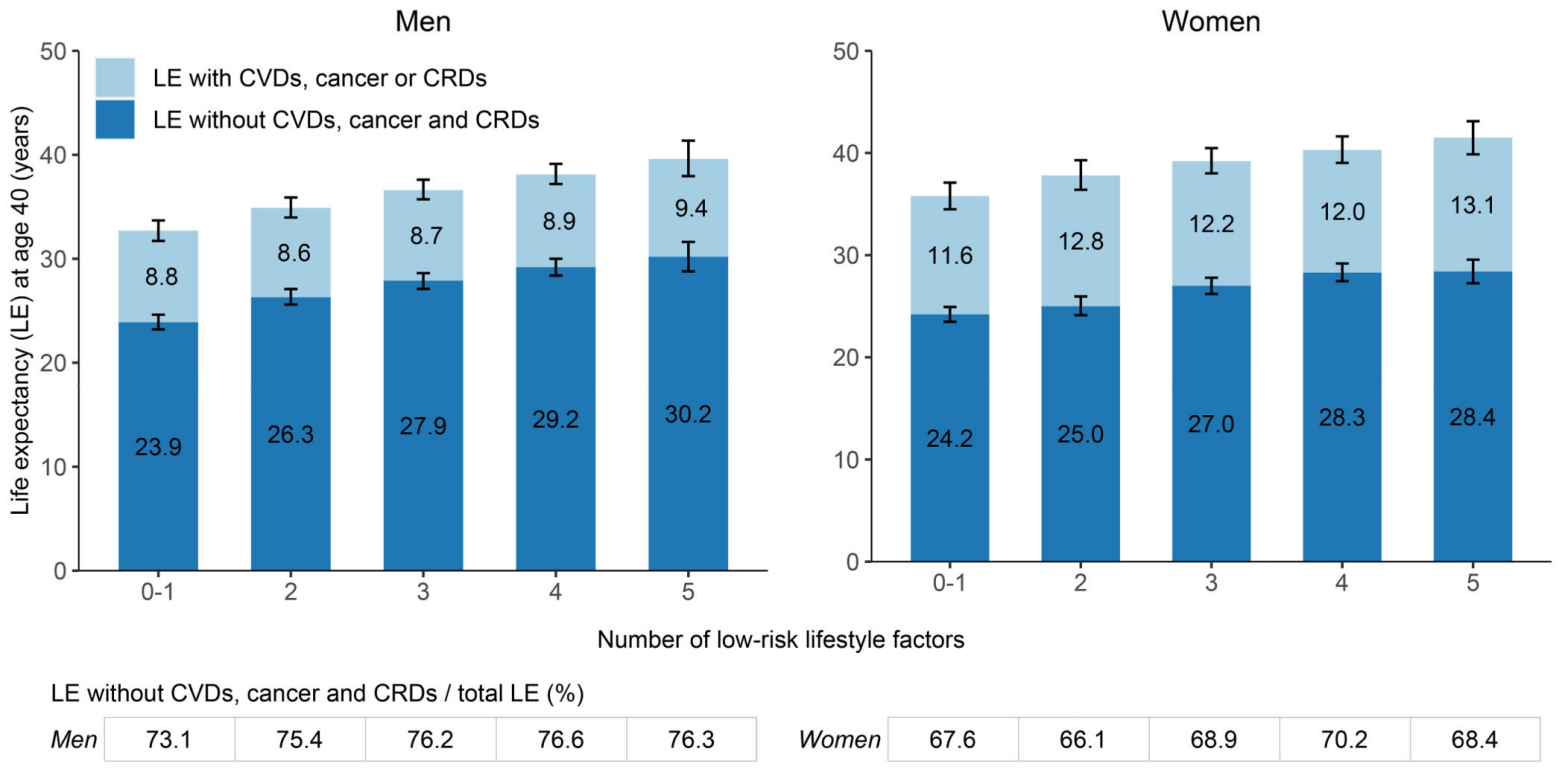
Life expectancy at age 40 years with and without cardiovascular diseases (CVDs), cancer, and/or chronic respiratory diseases (CRDs) by the number of low-risk lifestyle factors in men (n=181,544) and women (n=269,689) separately. The definition of low-risk lifestyle factors was the same as in Table 1. The error bars represent 95% CIs.

**Figure 4:**
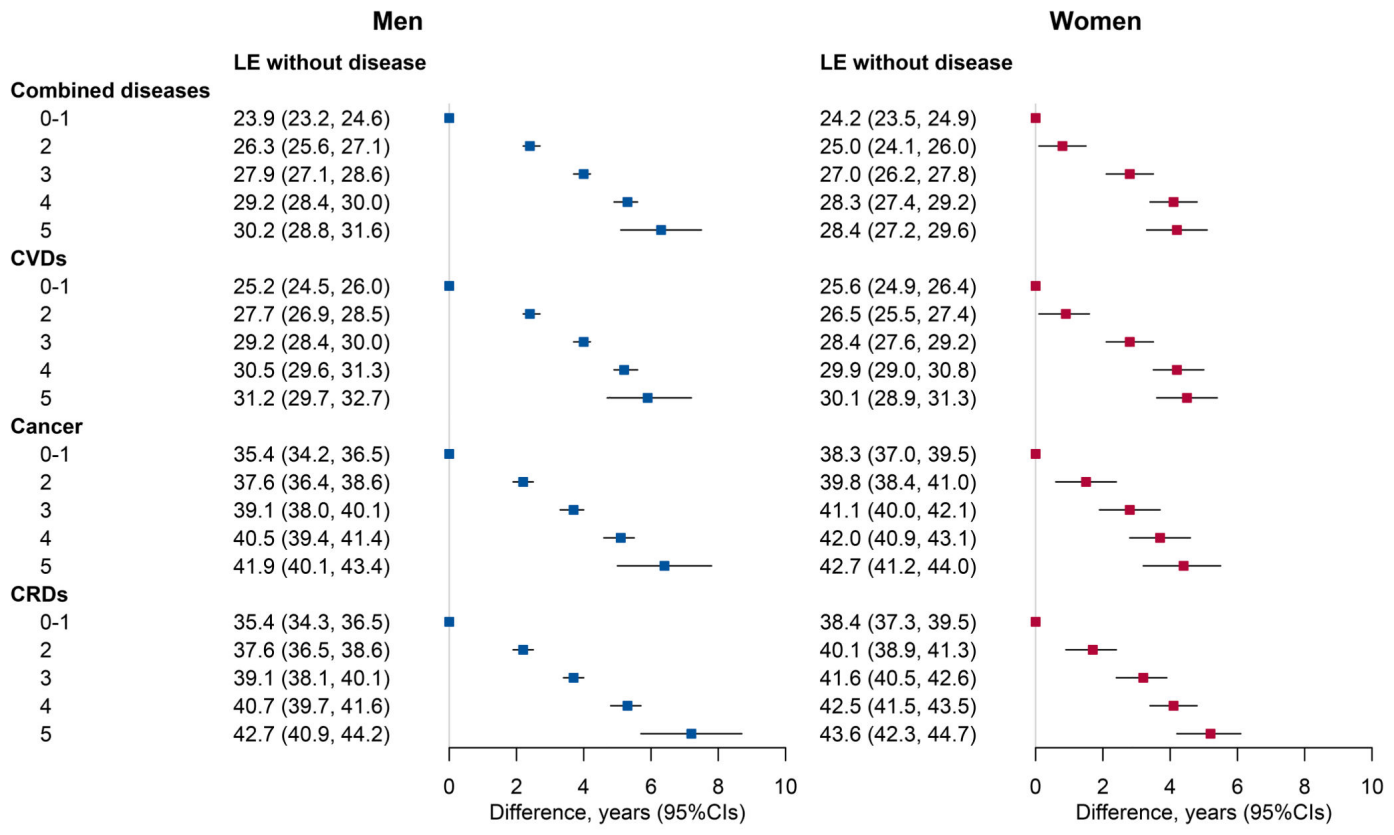
Life expectancy (LE) at age 40 years without chronic diseases and LE differences by the number of low-risk lifestyle factors in men (n=181,544) and women (n=269,689) separately. CVDs indicate cardiovascular diseases; CRDs, chronic respiratory diseases, including chronic obstructive pulmonary disease and asthma; CI, confidence interval. Combined diseases refer to a combination of CVDs, cancer, and CRDs, of which only the first occurrence was considered. The definition of low-risk lifestyle factors was the same as in Table 1. The error bars represent 95% CIs.

The joint analysis of five low-risk lifestyle factors showed that the total LE and LE free of the three NCDs, as well as the proportion of LE free of the three NCDs to the total LE, increased steadily with the increasing number of low-risk lifestyle factors (Figure 3). Women had a shorter LE free of the three NCDs but longer total LE and LE with the three NCDs than men. Sensitivity analyses showed the robustness of the results (Supplementary Figure 7). Similar results were observed at every age after age 40 (Supplementary Table 4 and Supplementary Figure 2). The LE free of the three NCDs (95%CI) at age 40 for individuals with 0-1 low-risk factor was on average 23.9 (23.2-24.6) years (73.1% of total LE) for men and 24.2 (23.5-24.9) years (67.6% of total LE) for women (Figure 4). When individuals adopted all five low-risk factors, it would reach 30.2 (28.8-31.6) years (76.3% of total LE) for men and 28.4 (27.2-29.6) years (68.4% of total LE) for women, with an increase of 6.3 (5.1-7.5) years (men) and 4.2 (3.6-5.4) years (women), respectively. In a sensitivity analysis using the low-risk score without healthy body fat levels, the estimated gains in disease-free LE associated with the other four low-risk lifestyle factors became much lower (Supplementary Figure 8).

In subgroup analyses, urban residents had similar LE free of the three NCDs to rural residents but longer total LE and LE with the NCDs than rural residents (Supplementary Figure 9). As the number of low-risk factors increased, urban residents gained more LE free of the NCDs than rural residents. When stratified by personal medical or family history, LE free of the three NCDs was lower for participants with a family history of any chronic diseases and for participants with hypertension or diabetes, but adopting low-risk lifestyles was associated with longer LE free of the three NCDs and higher share of total LE (Supplementary Figures 10 and 11). When we included T2D in the NCDs of interest, there was a slight decrease in LE free of the four NCDs, but the upward trend of LE free of the four NCDs persisted with the increasing number of low-risk lifestyle factors (Supplementary Figure 12).

When each disease was analyzed individually, the LE without cancer or CRDs was longer than without CVDs (Figure 4). The LE without CVDs was 6.0 (4.7-7.2) years (men) and 4.5 (3.6-5.4) years (women) longer in participants with all five low-risk lifestyle factors compared with those with 0-1 low-risk lifestyle factors. The corresponding extended years were 6.5 (5.0-7.8) years (men) and 4.4 (3.2-5.5) years for LE without cancer, and 7.3 (5.7-8.7) years (men) and 5.2 (4.2-6.1) years (women) for LE without CRDs.

## Discussion

In the present Chinese population, LE free of CVDs, cancer, and CRDs at age 40 accounted for about three-quarters of the residual LE in men and two-thirds in women (Supplementary Figure 2). The five low-risk lifestyle factors, namely never smoking or quitting for reasons other than illness, no excessive alcohol use, being physically active, healthy eating habits, and healthy body fat levels were associated with longer LE free of the three NCDs (Figure 2). When individuals adopted all five low-risk factors, their estimated LE free of the three NCDs at age 40 was on average 6.3 (95%CI: 5.1-7.5) years longer in men and 4.2 (3.6-5.4) years longer in women than those with 0-1 low-risk factors (Figure 3). Individuals with healthier lifestyles also showed the LE free of the three NCDs accounting for a slightly higher share of the total LE than those following unhealthy lifestyle choices.

A study based on the Nurses’ Health Study (NHS) and the Health Professionals Follow-Up Study (HPFS) found that the LE free of CVDs, cancer, and T2D at age 50 for individuals with 4-5 low-risk factors was on average 7.6 (95%CI: 6.8-8.4) years longer in men and 10.7 (10.0-11.3) years longer in women than those without any low-risk factors.^8^ The corresponding proportion of the disease-free LE to the total LE also increased from 75.3% to 79.0% in men and from 74.8% to 83.6% in women. In a pooled analysis of 12 European occupational cohorts, a healthy lifestyle was associated with an increase of LE free of T2D, coronary heart disease, stroke, cancer, asthma, and COPD at age 40 by 9.9 (6.7-13.1) years in men and 9.4 (5.4-13.3) years in women.^13^ The above two studies were performed in occupational populations or populations with high socioeconomic status and health levels. In contrast, our study quantified the associations of combined lifestyle factors with LE free of the major NCDs and its share of total LE in a Chinese population with diverse socio-demographic characteristics and from regions with different economic development levels. We observed that the proportion of the disease-free LE to the total LE increased with the number of low-risk factors, but the difference between the healthiest and the unhealthiest groups was lower than that in the fore mentioned studies.^8^

In the study of NHS and HPFS, a healthy lifestyle was associated with 8.6 years (for men) and 10.0 years (for women) increase in LE free of CVDs, and 6.0 years (for men) and 8.3 years (for women) increase in LE free of cancer at age 50.^8^ Whereas, in our study, the gains in LE free of CVDs at age 40 was 6.0 years in men and 4.5 years in women; the corresponding values for LE free of cancer was 6.5 years in men and 4.4 years in women. Possible explanations for the discrepancy in disease-free LE gains related to a healthy lifestyle include the differences in the characteristics of the study population and the definition of low-risk lifestyle factors. Other health risk factors important to the Chinese population are also worth noting. For instance, the Chinese population may expose to more environmental hazards in the living and working places, such as ambient and household air pollution, and soil and water pollution, possibly neutralizing the benefits of a healthy lifestyle. A meta-analysis has shown that the beneficial effects of physical activity on health might be attenuated under heavy air pollution.^16^ Moreover, the Chinese population generally had poor control of proximal risk factors like hypertension, dyslipidemia, and diabetes. They were all non-negligible contributors to the reduction of disease-free life years.^17^

In this study, the gain in disease-free LE, as well as its share of total LE, associated with a healthier lifestyle was bigger in men than in women, mainly because of the relatively stronger association between low-risk lifestyle factors and risks of chronic diseases and mortality. A possible explanation for this sex disparity could be that some non-traditional risk factors unique to or predominant in women may influence the relative strength of the association between lifestyle factors and outcomes, such as early menopause or menarche, gestational hypertension, and systemic inflammatory disorders.^18^ Previous studies also observed sex differences in the association between healthy lifestyle factors and disease-free LE but with inconsistent results, and the reason was unclear.^8,12,19^ It is also worth noting an important difference between urban and rural populations. A healthier lifestyle was associated with a greater increase in disease-free LE in urban than in rural populations (Supplementary Figure 9). The urban men showed a greater gain in disease-free LE than total LE, known as “absolute morbidity compression”.^3^

In the analyses of individual low-risk lifestyle factors, body fat levels defined jointly by BMI and WC had a smaller impact on total LE but a greater impact on disease-free LE than the other four lifestyle factors (Figure 2), corresponding to the relatively stronger associations of body fat levels with disease incidence than death (Figure 1). The obese individuals, despite a similar total LE to those with healthy body fat levels, spent more years living with diseases. This finding was consistent with most previous studies that used BMI as a single measure to define obesity and added to emphasize the importance of central obesity prevention for the Chinese population.^10,14,20^

Several strengths characterized this study. First, this study was based on a geographically spread Chinese population living in urban and rural areas. It filled the evidence gap of the association between a healthy lifestyle and disease-free LE in populations with less-developed economies and various socioeconomic characteristics. Second, the nature of the CKB study in terms of its sample size, long-term follow-up, and well-documented incident cases and deaths enables us to obtain reliable results within a single population following the unified study protocol. Third, the CKB study collected information on morbidity and mortality in continuous time by electronic linkage with local and national surveillance systems; thus, age-specific transition rates could be estimated and treated with the time-honored methods of the multistate life table.

This study also has limitations. First, the lifestyle factors were determined at baseline and not updated during the follow-up. However, among participants attending both the baseline survey and 2013-14 resurvey (a mean follow-up period of 8 years), most of them had not changed their risk level of lifestyles, regardless of disease occurrence during this period (Supplementary Table 2). Additionally, using baseline lifestyle factors can also avoid possible reverse causality resulting from lifestyle change after disease onset. Second, the CKB study used a qualitative food frequency questionnaire, limiting our analysis of dietary habits. For example, we defined the low-risk group of red meat intake as eating 1-6 days per week, without being able to consider the amount consumed. In some dietary contexts, red meat is considered unhealthy^22^ and there is evidence for a negative impact on health. Here, we consider red meat intake as an indicator of a healthy diet. This is because in underdeveloped rural areas, especially in the early 21st century, when the baseline survey of the CKB study was conducted, the food variety was not abundant, and the intake of nutritional supplements was rather low in the Chinese population.^38^ In this context, inadequate red meat intake might increase the risk of nutritional deficiency diseases.^21^ For this reason we consider a red meat intake of 1-6 days per week to indicate one part of a healthy diet. Third, the transition rates at every age resulted from a mixture of both the broad age range at entry and the long follow-up. Thus, cohort effects might impact our estimates. Fourth, the conclusion of this study was based on the premise that lifestyle factors have a causal association with the disease of interest, which, however, could not be inferred from the present study but supported by evidence from other Mendelian randomization studies.^23–25^

The present prospective cohort study of the Chinese population shows that a healthy lifestyle was associated with longer total LE and a larger proportion of remaining years lived without major NCDs, achieving “relative compression of morbidity”. The average LE of the Chinese population has reached the level of moderately developed countries.^1^ In addition to the goal of 79 years of LE at birth by 2030, the blueprint of Healthy China 2030 also calls for an increase in HLE. Public health initiatives for promoting healthy lifestyles, such as smoking restrictions and creating a supportive neighborhood environment for physical activity, would be critical to realizing this vision. Research on the impact of other health risk factors on HLE should also be enhanced to facilitate evidence-based policymaking to attain absolute compression of morbidity.

## Methods

### Study design and participants

The CKB study is a nationwide population-based prospective cohort study. Details of the study design have been described elsewhere.^26^ In brief, 512,725 participants aged 30-79 years were recruited from five urban and five rural areas in the 2004-08 baseline survey. Two periodic resurveys were conducted in 2008 and 2013-14 in a random sample of about 5% surviving participants. Information collected at baseline and resurveys were recorded using a laptop-based data entry system with built-in functions to avoid missing items and minimize logic errors. All participants signed an informed consent form. Ethical approval was obtained from the Ethics Review Committee of the Chinese Center for Disease Control and Prevention (CDC; Beijing, China) and the Oxford Tropical Research Ethics Committee, University of Oxford (UK).

For the current study, we excluded participants with prevalent coronary heart disease (n=15,472), stroke (n=8,884), cancer (n=2,578), COPD (n=37,057), or asthma (n=2,806) at baseline. Reasons for exclusion were not mutually exclusive, with 4,999 participants meeting multiple exclusion criteria. Two participants with missing values for BMI were excluded as well, leaving 451,233 participants in the primary analysis.

### Definition of low-risk lifestyle

Lifestyle-related factors were assessed by questionnaire and physical measurements at baseline. Details have been described in the Supplementary Material (appendix p2). Five modifiable lifestyle factors were compiled to create a gradient scale in our study: smoking, alcohol consumption, physical activity, dietary habits (fresh fruits, fresh vegetables, red meat, legumes, and fish), and body fat levels (BMI and WC, a reflection of balance between energy intake and energy expenditure).

The low-risk lifestyle for each factor was defined based on the Chinese dietary guidelines and related references.^8,27,28^ For smoking, participants that reported not smoking or quitting smoking for reasons other than illness were classified as low-risk group. For alcohol consumption, the low-risk group included non-regular drinkers and daily light-to-moderate drinkers (<30 g of pure alcohol in men and <15 g in women per day).^8,28^ Former smokers quitting due to illness and former drinkers were both excluded from the low-risk group to avoid the potential sick-quitter phenomenon.^29^ For physical activity, we defined those who engaged in an age- (<50, 50-59, and ≥60 years) and sex-specific median or higher level of physical activity as the low-risk group.^28^ For dietary habits, we derived a simple diet score based on the following criteria: eating fresh vegetables daily, eating fresh fruits daily, eating red meat 1-6 days per week, eating legumes ≥4 days per week, and eating fish ≥1 day per week. One point was scored for each criterion met, or zero. A score of 4 to 5 was classified as the low-risk group.^28,30^ We used the indicator of eating red meat 1-6 days per week, rather than considering different levels of red meat intake, because participants who ate red meat 4-6 days per week (HR=0.99 [95%CI: 0.97-1.02] for men; HR=0.97 [95%CI: 0.95-1.00] for women) showed similar risk of disease incidence as those who ate red meat 1-3 days per week (HR=0.97 [95%CI: 0.94-0.99] for men; HR=0.98 [95%CI: 0.96-1.00] for women). We recognize that red meat is associated with negative health impacts in other contexts^21,22^; however, we included red meat intake as an indicator of a healthy diet because red meat is rich in many nutrients like protein, iron, vitamin B_12_, and zinc,^30^ which were necessary for the Chinese population at a time when the food variety was not abundant and the intake of nutritional supplements was quite low.^38^ For body fat levels, both general and central adiposity indicators were taken into account to help distinguish between lean body mass and fat mass.^31^ Participants with a BMI of 18.5 to 27.9 kg/m^2^ and WC of <90/85 cm for men/women were defined as low-risk.^28^ The number of the low-risk lifestyle factors served as a simple score, ranging from 0 to 5, with higher scores indicating a healthier lifestyle.

### Ascertainment of disease onset and deaths

Participants were followed up for disease incidence and mortality since baseline recruitment via local disease and death registries and the national health insurance system, supplemented with annual active follow-up to minimize loss to follow-up. All events were coded by trained staff who were unaware of baseline information using the 10th revision of the International Classification of Diseases (ICD-10).

In the present study, CVDs and cancer were defined by codes I00-I99 and C00-C97, respectively. CRDs were defined jointly by COPD (J41-J44) and asthma (J45-J46). For T2D, we excluded cases clearly defined as insulin-dependent, malnutrition-related, or other specified diabetes (E10, E12, and E13), and defined T2D as code E11 or E14. Because the majority of the included participants were aged over 40 years, incident cases of unspecified diabetes (E14) were reasonably assumed as T2D.

Case adjudication for major chronic diseases has been ongoing since 2014. Qualified specialists blinded to baseline exposures of participants reviewed the medical records of incident cases. By October 2018, of 33,515 retrieved medical records of IHD cases and 40,465 retrieved medical records of stroke cases, the diagnosis was confirmed in 87.9% of IHD cases and 91.8% of stroke cases.^32^ Among the retrieved medical records of 18,581 cancer cases, 95.0% were confirmed for diagnosis. For COPD, the diagnosis was confirmed in 85% of the 1,069 retrieved medical records.^33^ Medical records of a random sample of 831 diabetes cases were also retrieved, and 98.6% were confirmed for diagnosis.

### Statistical analysis

We analyzed the changes in dichotomized lifestyle factors between the 2004-08 baseline and 2013-14 resurvey and whether such changes differed by the occurrence of any of the three NCDs, with adjustment for age, sex, and study area, as appropriate.

To calculate life expectancies in different health states, we applied the population-based multistate life table (MSLT) method, which is a demographic tool incorporating both the morbidity and mortality experience of multiple birth cohorts during a certain follow-up period.^34^ We considered three states in this study: disease-free, presence of disease, and death. Possible transition directions were: (1) from disease-free to the presence of disease; (2) from disease-free to death without experiencing the disease, and (3) from the presence of disease to all-cause mortality. No backflows were allowed, and only the first entry into a state was considered. ^8,35^ For participants who died on the same date of the first disease diagnosis, we calculated the date of disease onset as the date of death minus 0.5 days in the primary analysis.^32^

Due to the sex differences in LE, we performed all analyses for men and women separately. We built MSLTs separately for CVDs, cancer, CRDs, and the combination of these three diseases, following a similar approach to previous studies.^8,12,19^ First, we calculated the observed transition rates by dividing the number of events by the corresponding risk period of exposure in each state for every single year of age. For smoothing the observed age-specific transition rates, we applied Poisson regression with attained age as the only covariate.^19^ Second, we calculated the prevalence of low-risk lifestyle factors by 5-year age (attained age) groups separately for participants with and without the diseases, assuming that the baseline assessment reflected the lifestyle within the follow-up period. Subsequently, we estimated the sex-specific HRs of the association between lifestyle factors and each transition by using separate Cox proportional hazard models with age as the time scale. When modeling the hazard of transition from the presence of disease to all-cause mortality, we used the time after disease onset as the time scale, with age at disease onset as a fixed covariate.^36^ We assessed the proportional hazards assumption by plotting log-log survival estimates against time, and all Cox regression models satisfied the assumption. The Cox model was stratified jointly by 10 study areas and age at baseline in a 5-year interval, with adjustment for education level, marital status, menopausal status (women only), and family histories of heart attack, stroke, or cancer. Lastly, transition rates of each category of lifestyle factor were calculated separately by integrating the foregoing results. To avoid unstable rates due to the limited number of events under age 40, MSLTs started at age 40 and closed at age 90 since few participants reached this age during the follow-up period.

We conducted several sensitivity analyses. We estimated the HRs after excluding participants who experienced disease onset or died within the first two years of follow-up to minimize potential reverse causality. We additionally adjusted for hypertension, diabetes, and usage of antihypertensive, glucose-lowering medications, and statin at baseline. For participants who died on the same date of disease onset, we considered several other alternatives to repeat all analyses: (□) calculating the date of disease onset using different time intervals (0.5 and 1 year); (□) regarding them as death without disease onset; (□) excluding these participants. To evaluate the impact of the traditional lifestyle factors without body fat levels, a low-risk lifestyle score was also created based on the other four low-risk factors.

Subgroup analyses were performed by residence (urban or rural), family history of chronic diseases (with a family history of heart attack, stroke, or cancer, or none), and baseline disease status (with hypertension or diabetes, or none). Also, we repeated the primary analysis with an alternative outcome that included T2D in addition to the three diseases. In this analysis, participants with diabetes at baseline were further excluded (n=24,134).

All statistical analyses were performed using Stata (version 15.0, StataCorp). The CIs for LE was estimated using @RISK 8.1 (Palisade Corp, Ithaca, NY), with 10,000 runs of Monte Carlo simulation (parametric bootstrapping).^37^ Graphs were plotted using R version 4.0.3. All *P* values shown in this study were two-sided.

## Supplementary Material

Supplementary Material

## Figures and Tables

**Table 1:** Multivariable-adjusted hazard ratios (95% CIs) for each transition by the number of low-risk lifestyle factors in men and women separately.

	Number of events *	Events/PY s (/1,000)	HRs (95% CIs)						*P* for trend
			0-1	2	3	4	5	Per 1-factor increase	
**Men (n=181,544)**									
Baseline → disease									
CVDs, cancer or CRDs	55,757	31.9	1.00 (Referent)	0.80 (0.78-0.81)	0.69 (0.67-0.71)	0.61 (0.59-0.63)	0.56 (0.50-0.63)	0.85 (0.84-0.86)	<0.001
CVDs	44,970	25.3	1.00 (Referent)	0.80 (0.78-0.82)	0.69 (0.68-0.71)	0.63 (0.61-0.65)	0.60 (0.53-0.68)	0.85 (0.85-0.86)	<0.001
Cancer	11,816	6.1	1.00 (Referent)	0.80 (0.76-0.84)	0.70 (0.67-0.74)	0.58 (0.54-0.62)	0.50 (0.38-0.64)	0.84 (0.82-0.86)	<0.001
CRDs	5,956	3.1	1.00 (Referent)	0.77 (0.72-0.82)	0.63 (0.59-0.68)	0.49 (0.44-0.55)	0.29 (0.17-0.50)	0.79 (0.77-0.81)	<0.001
Baseline → death^†^									
CVDs, cancer or CRDs	2,674	1.5	1.00 (Referent)	0.80 (0.72-0.89)	0.73 (0.65-0.81)	0.64 (0.55-0.74)	0.40 (0.20-0.80)	0.86 (0.83-0.90)	<0.001
CVDs	8,394	4.7	1.00 (Referent)	0.79 (0.75-0.84)	0.68 (0.64-0.72)	0.55 (0.50-0.60)	0.43 (0.30-0.61)	0.83 (0.81-0.85)	<0.001
Cancer	11,603	6.0	1.00 (Referent)	0.78 (0.74-0.82)	0.63 (0.60-0.66)	0.54 (0.50-0.58)	0.43 (0.31-0.58)	0.81 (0.79-0.82)	<0.001
CRDs	17,388	9.0	1.00 (Referent)	0.79 (0.75-0.82)	0.66 (0.63-0.69)	0.55 (0.52-0.58)	0.44 (0.34-0.56)	0.82 (0.81-0.83)	<0.001
Disease → death^‡^									
CVDs, cancer or CRDs	16,481	81.2	1.00 (Referent)	0.92 (0.88-0.96)	0.83 (0.79-0.86)	0.74 (0.69-0.78)	0.64 (0.50-0.83)	0.90 (0.89-0.92)	<0.001
CVDs	10,761	62.2	1.00 (Referent)	0.88 (0.84-0.93)	0.77 (0.73-0.81)	0.69 (0.64-0.75)	0.58 (0.43-0.79)	0.88 (0.86-0.90)	<0.001
Cancer	7,552	307.4	1.00 (Referent)	0.95 (0.89-1.01)	0.88 (0.82-0.94)	0.82 (0.75-0.90)	0.87 (0.61-1.25)	0.94 (0.91-0.96)	<0.001
CRDs	1,767	98.7	1.00 (Referent)	0.86 (0.77-0.98)	0.73 (0.64-0.83)	0.63 (0.50-0.78)	1.00 (0.40-2.47)	0.86 (0.81-0.91)	<0.001
**Women (n=269,689)**									
Baseline → disease									
CVDs, cancer or CRDs	77,821	29.3	1.00 (Referent)	0.93 (0.87-0.99)	0.78 (0.73-0.83)	0.69 (0.65-0.74)	0.69 (0.64-0.75)	0.88 (0.87-0.89)	<0.001
CVDs	66,032	24.5	1.00 (Referent)	0.93 (0.87-1.00)	0.78 (0.73-0.83)	0.68 (0.64-0.73)	0.67 (0.62-0.73)	0.87 (0.86-0.88)	<0.001
Cancer	12,819	4.4	1.00 (Referent)	0.94 (0.79-1.12)	0.87 (0.73-1.03)	0.85 (0.72-1.01)	0.85 (0.69-1.03)	0.96 (0.94-0.98)	<0.001
CRDs	6,550	2.2	1.00 (Referent)	0.81 (0.70-0.94)	0.64 (0.55-0.74)	0.57 (0.49-0.67)	0.47 (0.36-0.61)	0.84 (0.81-0.86)	<0.001
Baseline → death^†^									
CVDs, cancer or CRDs	2,036	0.8	1.00 (Referent)	0.97 (0.68-1.40)	0.81 (0.57-1.16)	0.65 (0.45-0.94)	0.57 (0.34-0.94)	0.83 (0.78-0.88)	<0.001
CVDs	6,261	2.3	1.00 (Referent)	0.87 (0.70-1.07)	0.78 (0.63-0.96)	0.71 (0.58-0.88)	0.58 (0.43-0.76)	0.90 (0.87-0.93)	<0.001
Cancer	9,945	3.4	1.00 (Referent)	0.75 (0.66-0.85)	0.59 (0.51-0.67)	0.45 (0.39-0.51)	0.36 (0.28-0.45)	0.77 (0.75-0.79)	<0.001
CRDs	14,430	4.9	1.00 (Referent)	0.81 (0.72-0.92)	0.67 (0.60-0.76)	0.58 (0.51-0.66)	0.50 (0.41-0.59)	0.84 (0.83-0.86)	<0.001
Disease → death^‡^									
CVDs, cancer or CRDs	13,549	42.7	1.00 (Referent)	0.78 (0.69-0.88)	0.74 (0.66-0.83)	0.69 (0.62-0.78)	0.57 (0.47-0.69)	0.93 (0.91-0.95)	<0.001
CVDs	9,324	34.2	1.00 (Referent)	0.76 (0.67-0.87)	0.69 (0.60-0.78)	0.59 (0.52-0.68)	0.51 (0.40-0.64)	0.87 (0.85-0.90)	<0.001
Cancer	5,640	146.4	1.00 (Referent)	0.96 (0.77-1.19)	0.97 (0.78-1.20)	0.93 (0.74-1.15)	0.84 (0.63-1.11)	0.97 (0.94-1.01)	0.142
CRDs	1,155	48.9	1.00 (Referent)	0.64 (0.48-0.85)	0.66 (0.50-0.87)	0.47 (0.35-0.64)	0.35 (0.15-0.83)	0.84 (0.79-0.91)	<0.001

CVDs indicate cardiovascular diseases; CRDs, chronic respiratory diseases, including chronic obstructive pulmonary disease and asthma; PYs, person-years; HR, hazard ratio; CI, confidence interval.

Cox regression was used to estimate the HRs and 95% CIs, with adjustment for education (no formal school, primary school, middle school, high school, college, or university or higher), marital status (married, widowed, divorced or separated, or never married), family histories of heart attack, stroke, and cancer (presence, absence, or unknown), and menopausal status (women only) as appropriate. In the analysis of transitions from a disease state to all-cause mortality, HRs were further adjusted for age at diagnosis of corresponding diseases (years). All statistical tests were two-sided.

Low-risk lifestyle factors were defined as: never smoking or having stopped for reasons other than illness; less than daily drinking or drinking <30 g (men)/15 g (women) of pure alcohol per day (former drinkers excluded); engaging in an age- (<50 years, 50-59 years, and ≥60 years) and sex-specific median or higher level of physical activity; having at least 4 of the following dietary habits: eating fresh vegetables daily, eating fresh fruits daily, eating red meat 1-6 days per week, eating legumes ≥4 days per week, eating fish ≥1 day per week; having a BMI between 18.5 and 27.9 kg/m and a waist circumference <90 cm (men)/85 cm (women).

* Number of events refers to the number of cases in each transition.

† The transition from baseline to death refers to the transition from a disease-free state at baseline to death from any cause other than the concerned disease without experiencing disease onset.

‡ The transition from disease to death refers to the transition from the concerned disease state to all-cause death.

## Data Availability

China Kadoorie Biobank data are available to all bona fide researchers. Details of how to access and details of the data release schedule are available from www.ckbiobank.org/site/Data+Access. As stated in the access policy, the CKB study group must maintain the integrity of the database for future use and regulate data access to comply with prior conditions agreed with the Chinese government. Data security is an integral part of CKB study protocols. Data can be released outside the CKB research group only with appropriate security safeguards.

## Code availability statement

Analysis code for this study is available at https://github.com/qiufen-code/Lifestyle-and-disease-free-LE.
