# Supplementary material for: Healthy lifestyle and life expectancy free of major chronic diseases at age 40 in China
Source: Nat Hum Behav. Author manuscript; Available in PMC 2023 Sep 23. (PMC7615116; doi:10.1038/s41562-023-01624-7)
Supplement: Supplementary Material [file EMS184944-supplement-Supplementary_Material.pdf]

nature human behaviour

Article

https://doi.org/10.1038/s41562-023-01624-7

Healthy lifestyle and life expectancy free of major chronic diseases at age 40 in China

In the format provided by the authors and unedited

Supplementary Material

Members of the China Kadoorie Biobank collaborative group 1

Assessment of lifestyle factors and other covariates in China Kadoorie Biobank (CKB) study.. 2

References 3

Supplementary Table 1. Baseline characteristics of the study participants according to the number of low-risk lifestyle factors 4

Supplementary Table 2. Changes in lifestyle factors between 2004-08 baseline and 2013-14 resurvey by chronic disease status at 2013-14 resurvey in 22 275 participants 7

Supplementary Table 3. Sensitivity analysis of associations of the number of low-risk lifestyle factors with each transition in men and women separately. 8

Supplementary Table 4. Life expectancy at ages 40, 50, and 65 years without chronic diseases and life expectancy differences by the number of low-risk lifestyle factors in men and women separately. 12

Supplementary Figure 1. Observed and predicted transition rates of CKB participants in men (n=181,544) and women (n=269,689) separately. 13

Supplementary Figure 2. Life expectancy at every age after age 40 years with and without cardiovascular diseases (CVDs), cancer, and/or chronic respiratory diseases (CRDs), conditional on disease-free at age 40 years and by the number of low-risk lifestyle factors in men (n=181,544) and women (n=269,689) separately. 14

Supplementary Figure 3. Projected gained or lost life expectancy (LE) at age 40 years without cardiovascular diseases (CVDs), cancer, and chronic respiratory diseases (CRDs) by levels of individual lifestyle risk factors in men (n=181,544) and women (n=269,689) separately. 15

Supplementary Figure 4. Life expectancy at age 40 years with and without cardiovascular diseases (CVDs) by levels of individual lifestyle risk factors in men (n=181,544) and women (n=269,689) separately. 17

Supplementary Figure 5. Life expectancy at age 40 years with and without cancer by levels of individual lifestyle risk factors in men (n=181,544) and women (n=269,689) separately. 18

Supplementary Figure 6. Life expectancy at age 40 years with and without chronic respiratory diseases (CRDs) by levels of individual lifestyle risk factors in men (n=181,544) and women (n=269,689) separately. 19

Supplementary Figure 7. Sensitivity analysis of life expectancy at age 40 years with and without cardiovascular diseases (CVDs), cancer, and/or chronic respiratory diseases (CRDs) by the number of low-risk lifestyle factors. 20

Supplementary Figure 8. Sensitivity analysis of life expectancy at age 40 years with and without cardiovascular diseases (CVDs), cancer, and/or chronic respiratory diseases (CRDs) by the number of low-risk lifestyle factors without considering body fat levels in men (n=181,544) and women (n=269,689) separately. 22

Supplementary Figure 9. Life expectancy at age 40 years with and without cardiovascular diseases (CVDs), cancer, and/or chronic respiratory diseases (CRDs) by the number of low-risk lifestyle factors stratified by residence. 23

Supplementary Figure 10. Life expectancy at age 40 years with and without cardiovascular diseases (CVDs), cancer, and/or chronic respiratory diseases (CRDs) by the number of low-risk lifestyle factors stratified by family history of chronic diseases. 24

Supplementary Figure 11. Life expectancy at age 40 years with and without cardiovascular diseases (CVDs), cancer, and/or chronic respiratory diseases (CRDs) by the number of low-risk lifestyle factors stratified by prevalent hypertension and/or type 2 diabetes (T2D) at baseline. 25

Supplementary Figure 12. Life expectancy at age 40 years with and without cardiovascular diseases (CVDs), cancer, chronic respiratory diseases (CRDs), and/or type 2 diabetes (T2D) by the number of low-risk lifestyle factors in men (n=172,292) and women (n=254,807) separately. 26

Members of the China Kadoorie Biobank collaborative group

International Steering Committee: Junshi Chen, Zhengming Chen (PI), Robert Clarke, Rory Collins, Yu Guo, Liming Li (PI), Jun Lv, Richard Peto, Robin Walters. **International Co-ordinating Centre, Oxford:** Daniel Avery, Derrick Bennett, Ruth Boxall, Sue Burgess, Ka Hung Chan, Yumei Chang, Yiping Chen, Zhengming Chen, Johnathan Clarke; Robert Clarke, Huaidong Du, Ahmed Edris Mohamed, Zammy Fairhurst-Hunter, Hannah Fry, Simon Gilbert, Alex Hacker, Mike Hill, Michael Holmes, Pek Kei Im, Andri Iona, Maria Kakkoura, Christiana Kartsonaki, Rene Kerosi, Kuang Lin, Mohsen Mazidi, Iona Millwood, Sam Morris, Qunhua Nie, Alfred Pozarickij, Paul Ryder, Saredo Said, Sam Sansome, Dan Schmidt, Paul Sherliker, Rajani Sohoni, Becky Stevens, Iain Turnbull, Robin Walters, Lin Wang, Neil Wright, Ling Yang, Xiaoming Yang, Pang Yao.
National Co-ordinating Centre, Beijing: Yu Guo, Xiao Han, Can Hou, Jun Lv, Pei Pei, Chao Liu, Canqing Yu, Qingmei Xia. **10 Regional Co-ordinating Centres: Qingdao CDC:** Zengchang Pang, Ruqin Gao, Shanpeng Li, Haiping Duan, Shaojie Wang, Yongmei Liu, Ranran Du, Yajing Zang, Liang Cheng, Xiaocao Tian, Hua Zhang, Yaoming Zhai, Feng Ning, Xiaohui Sun, Feifei Li. **Licang CDC:** Silu Lv, Junzheng Wang, Wei Hou. **Heilongjiang Provincial CDC:** Wei Sun, Shichun Yan, Xiaoming Cui. **Nangang CDC:** Chi Wang, Zhenyuan Wu,Yanjie Li, Quan Kang. **Hainan Provincial CDC:** Huiming Luo, Tingting Ou. **Meilan CDC:** Xiangyang Zheng, Zhendong Guo, Shukuan Wu, Yilei Li, Huimei Li. **Jiangsu Provincial CDC:** Ming Wu, Yonglin Zhou, Jinyi Zhou, Ran Tao, Jie Yang, Jian Su. **Suzhou CDC:** Fang Liu, Jun Zhang, Yihe Hu, Yan Lu, Liangcai Ma, Aiyu Tang, Shuo Zhang, Jianrong Jin, Jingchao Liu. **Guangxi Provincial CDC:** Mei Lin, Zhenzhen Lu. **Liuzhou CDC:** Lifang Zhou, Changping Xie, Jian Lan,Tingping Zhu,Yun Liu, Liuping Wei, Liyuan Zhou, Ningyu Chen, Yulu Qin, Sisi Wang. **Sichuan Provincial CDC:** Xianping Wu, Ningmei Zhang, Xiaofang Chen, Xiaoyu Chang. **Pengzhou CDC:** Mingqiang Yuan, Xia Wu, Xiaofang Chen, Wei Jiang, Jiaqiu Liu, Qiang Sun. **Gansu Provincial CDC:** Faqing Chen, Xiaolan Ren, Caixia Dong. **Maiji CDC:** Hui Zhang, Enke Mao, Xiaoping Wang, Tao Wang, Xi zhang. **Henan Provincial CDC:** Kai Kang, Shixian Feng, Huizi Tian, Lei Fan. **Huixian CDC:** XiaoLin Li, Huarong Sun, Pan He, Xukui Zhang. **Zhejiang Provincial CDC:** Min Yu, Ruying Hu, Hao Wang. **Tongxiang CDC**: Xiaoyi Zhang, Yuan Cao, Kaixu Xie, Lingli Chen, Dun Shen. **Hunan Provincial CDC:** Xiaojun Li, Donghui Jin, Li Yin, Huilin Liu, Zhongxi Fu. **Liuyang CDC:** Xin Xu, Hao Zhang, Jianwei Chen,Yuan Peng, Libo Zhang, Chan Qu.

Assessment of lifestyle factors and other covariates in China Kadoorie Biobank (CKB) study

Lifestyle-related factors were assessed by questionnaire and physical measurements. Ever smokers were asked to report the frequency, type, and the amount of tobacco smoked per day. Former smokers were additionally asked for years since stopping and the reason for quitting smoking. Participants who reported drinking at least once a week were asked for the number of drinking days per week, the type of alcoholic beverage consumed habitually and the amount drunk on a typical drinking day in the past 12 months. For physical activity, participants were asked for the usual type and duration of occupational, commuting, domestic, and leisure-time-related activities in the past 12 months. The daily level of physical activity was calculated by multiplying the metabolic equivalent of task (MET) value of each type of activity and the hours spent on that activity per day and then summing the MET-hours for all activities.[1] Eating habits of 12 conventional food groups in the past 12 months were assessed using a validated food frequency questionnaire (FFQ).[2]

In a subsample of 17,355 participants who completed the same questionnaire twice at a median interval of 2.6 years, we observed moderate to excellent reproducibility for most of the lifestyle variables. The weighted kappa coefficient was 0.87 for tobacco smoking and 0.64 for alcohol intake. The Spearman correlation coefficient was 0.55 for physical activity level. For food items from the qualitative FFQ, the weighted kappa coefficients ranged from 0.61-0.88, except for fresh vegetables, the reproducibility of which may be influenced by seasonal availability.[3,4]

Weight, height, and waist circumference (WC) were measured by trained staff using calibrated instruments. BMI was calculated as weight in kilograms divided by height in meters squared.

References

1. Du H, Bennett D, Li L, et al. Physical activity and sedentary leisure time and their associations with BMI, waist circumference, and percentage body fat in 0.5 million adults: the China Kadoorie Biobank study. *Am J Clin Nutr* 2013; **97**(3): 487-96.
2. Zhu N, Yu C, Guo Y, et al. Adherence to a healthy lifestyle and all-cause and cause-specific mortality in Chinese adults: a 10-year prospective study of 0.5 million people. *The international journal of behavioral nutrition and physical activity* 2019; **16**(1): 98.
3. Qin C, Guo Y, Pei P, et al. The Relative Validity and Reproducibility of Food Frequency Questionnaires in the China Kadoorie Biobank Study. *Nutrients* 2022; **14**(4).
4. Lv J, Yu C, Guo Y, et al. Adherence to Healthy Lifestyle and Cardiovascular Diseases in the Chinese Population. *Journal of the American College of Cardiology* 2017; **69**(9): 1116-25.

Supplementary Table 1. Baseline characteristics of the study participants according to the number of low-risk lifestyle factors

	Number of low-risk lifestyle factors†				
	0-1	2	3	4	5
**Men (n =181,544)**					
No. of participants, n (%)*	32,241 (17.8)	64,246 (35.4)	63,388 (34.9)	20,374 (11.2)	1,295 (0.7)
Age, year (SD)	52.0 (10.3)	51.7 (10.4)	51.4 (10.7)	51.3 (10.9)	52.2 (11.2)
Urban area, n (%)	16,027 (49.7)	28,007 (43.6)	25,146 (39.7)	8,784 (43.1)	904 (69.8)
Middle school and above, n (%)	20,193 (59.4)	38,069 (59.2)	36,804 (59.7)	12,723 (62.9)	957 (70.0)
Married, n (%)	30,119 (93.4)	60,056 (93.5)	59,093 (93.2)	19,081 (93.5)	1,240 (95.3)
Lifestyle-related factors					
Non-smoking, n (%)	1,557 (4.3)	11,846 (18.1)	25,926 (42.9)	18,546 (91.7)	-
Non-excessive alcohol intake, n (%)	11,590 (39.1)	46,289 (72.6)	59,267 (93.1)	20,130 (98.8)	-
Physical activity, MET-h/d	14.5 (9.9)	20.1 (14.2)	27.1 (15.5)	31.7 (13.9)	31.3 (12.5)
Dietary metrics, n (%)					
Eating vegetables daily	31,090 (94.7)	61,144 (94.7)	59,303 (94.8)	19,245 (95.0)	-
Eating fruits daily	4,135 (10.5)	8,074 (12.6)	8,563 (15.1)	4,385 (21.9)	-
Eating red meat 1-6 days/week	15,015 (47.4)	33,134 (50.9)	33,909 (53.5)	11,839 (58.7)	-
Eating fish ≥1 day/week	17,161 (50.9)	31,875 (49.2)	29,686 (49.0)	10,765 (52.3)	-
Eating legumes ≥4 days/week	2,794 (8.5)	5,417 (8.6)	6,413 (10.3)	3,769 (17.8)	-
Body mass index, kg/m^2	24.7 (3.9)	23.6 (3.3)	23.0 (2.7)	22.9 (2.3)	22.7 (2.2)
Waist circumference, cm	87.0 (11.4)	82.5 (9.9)	80.1 (8.0)	79.5 (6.9)	79.0 (6.4)
Prevalent hypertension, n (%)	14,185 (44.4)	22,753 (35.6)	20,363 (32.0)	6,489 (31.2)	411 (28.9)
Prevalent diabetes, n (%)	2,512 (7.2)	3,339 (5.2)	2,537 (4.2)	810 (4.0)	54 (3.2)
Family history of, n (%)					
Heart attack	1,233 (3.6)	1,962 (3.1)	1,925 (3.1)	622 (3.1)	38 (2.6)
Stroke	6,312 (19.1)	11,576 (18.0)	10,925 (17.4)	3,577 (17.6)	212 (16.7)
Cancer	5,948 (17.9)	11,001 (17.0)	10,300 (16.6)	3,292 (16.2)	220 (16.3)
**Women (n = 269,689)**					

	Number of low-risk lifestyle factors†				
	0-1	2	3	4	5
No. of participants, n (%)*	1,940 (0.7)	43,168 (16.0)	118,911 (44.1)	97,718 (36.2)	7,952 (3.0)
Age, year (SD)	58.7 (10.3)	54.2 (10.3)	50.6 (10.2)	49.1 (9.7)	48.7 (9.6)
Urban area, n (%)	820 (42.3)	20,692 (47.9)	52,398 (44.1)	38,402 (39.3)	5,545 (69.7)
Middle school and above, n (%)	554 (34.1)	17,252 (40.1)	55,588 (44.4)	40,645 (45.3)	5,233 (56.3)
Married, n (%)	1,461 (86.7)	37,127 (89.4)	106,516 (89.6)	89,908 (90.5)	7,296 (91.0)
Lifestyle-related factors					
Non-smoking, n (%)	446 (51.2)	40,292 (93.7)	116,981 (98.3)	97,643 (99.9)	-
Non-excessive alcohol intake, n (%)	1,039 (72.2)	41,520 (95.8)	117,752 (99.0)	97,632 (99.9)	-
Physical activity, MET-h/d	14.3 (7.0)	13.8 (5.4)	17.5 (10.6)	27.7 (12.2)	29.5 (11.2)
Dietary metrics, n (%)					
Eating vegetables daily	1,872 (93.6)	41,499 (94.8)	112,938 (94.6)	90,690 (94.3)	-
Eating fruits daily	390 (15.1)	7,927 (16.4)	22,959 (19.4)	20,609 (23.2)	-
Eating red meat 1-6 days/week	976 (45.2)	19,800 (48.9)	60,189 (51.2)	57,922 (57.5)	-
Eating fish ≥1 day/week	520 (39.0)	18,051 (41.8)	52,560 (43.9)	43,910 (46.5)	-
Eating legumes ≥4 days/week	120 (5.9)	2,003 (4.7)	7,223 (6.3)	10,963 (11.0)	-
Body mass index, kg/m^2	25.4 (4.6)	26.2 (4.3)	23.8 (3.4)	22.8 (2.3)	22.7 (2.1)
Waist circumference, cm	85.3 (11.7)	86.5 (10.6)	79.0 (9.2)	75.5 (6.3)	75.1 (5.7)
Prevalent hypertension, n (%)	865 (36.2)	19,522 (40.2)	37,448 (32.1)	26,075 (27.8)	1,996 (27.5)
Prevalent diabetes, n (%)	244 (8.3)	4,486 (8.5)	6,428 (5.5)	3,438 (3.9)	286 (3.6)
Family history of, n (%)					
Heart attack	48 (2.4)	1,434 (3.2)	3,853 (3.2)	2,953 (3.2)	357 (3.9)
Stroke	372 (17.2)	8,612 (17.9)	21,549 (17.7)	15,590 (17.4)	1,455 (18.0)
Cancer	373 (18.3)	7,770 (16.3)	19,646 (16.4)	15,039 (16.4)	1,443 (16.8)
Postmenopausal, n (%)	1,513 (51.0)	27,382 (50.0)	58,591 (49.5)	42,665 (49.1)	3,142 (48.7)

MET-h/d indicates metabolic equivalent of tasks hours/day.

All variables were presented as mean (standard deviation) or number (percentage). Baseline characteristics were adjusted for age and study area, except in the cases where age or study area was the independent variable.

[*]The numbers in parentheses indicate the proportion of participants who had a different number of low-risk lifestyle factors.

[†]Low-risk lifestyle factors were defined as: never smoking or having stopped for reasons other than illness; less than daily drinking or drinking <30 g (men)/15 g (women) of pure alcohol per day (former drinkers excluded); engaging in an age- (<50 years, 50-59 years, and ≥60 years) and sex-specific median or higher level of physical activity; having at least 4 of the following dietary habits: eating fresh vegetables daily, eating fresh fruits daily, eating red meat 1-6 days per week, eating legumes ≥4 days per week, eating fish ≥1 day per week; having a BMI between 18.5 and 27.9 kg/m^2 and a waist circumference <90 cm (men)/85 cm (women).

Supplementary Table 2. Changes in lifestyle factors between 2004-08 baseline and 2013-14 resurvey by chronic disease status at 2013-14 resurvey in 22 275 participants

	No concerned diseases*	With at least one of the concerned diseases*	Total
No. of participants, n	19,151	3,124	22,275
Age at baseline, years	49.9 (9.7)	55.9 (9.6)	50.7 (9.9)
Age at resurvey, years	57.9 (9.7)	64.0 (9.6)	58.7 (9.9)
Physical activity, MET-h/d†	-3.1 (16.5)	-4.3 (14.2)	-3.3 (16.2)
Smoking			
Stable	93.7 (93.4-94.0)	91.0 (90.0-91.9)	93.3 (93.0-93.6)
Worse	1.4 (1.2-1.6)	1.3 (0.9-1.7)	1.4 (1.2-1.5)
Better	4.9 (4.6-5.2)	7.7 (6.8-8.6)	5.3 (5.0-5.6)
Alcohol intake			
Stable	90.9 (90.5-91.3)	89.8 (88.8-90.8)	90.7 (90.3-91.1)
Worse	5.2 (4.9-5.5)	5.3 (4.5-6.0)	5.2 (4.9-5.5)
Better	3.9 (3.7-4.2)	4.9 (4.2-5.7)	4.1 (3.8-4.4)
Diet score			
Stable	87.8 (87.4-88.3)	88.2 (87.1-89.3)	87.9 (87.5-88.3)
Worse	6.0 (5.7-6.4)	5.6 (4.9-6.4)	6.0 (5.7-6.3)
Better	6.1 (5.8-6.5)	6.1 (5.3-7.0)	6.1 (5.8-6.5)
Body fat levels			
Stable	73.5 (72.9-74.2)	73.5 (71.9-75.1)	73.5 (73.0-74.1)
Worse	22.1 (21.6-22.7)	22.2 (20.6-23.7)	22.2 (21.6-22.7)
Better	4.3 (4.0-4.6)	4.3 (3.6-5.0)	4.3 (4.1-4.6)

MET-h/d indicates metabolic equivalent of task hours per day.
All variables were adjusted for age at enrollment, sex, and study area, as appropriate.
Age and physical activity are presented as means (standard deviations). The others are presented as percentages (95% confidence intervals).
The definition of low-risk lifestyle factors was the same as in Supplementary Table 1.
Changes in lifestyle factors between baseline and resurvey were classified into stable (at the same risk level), worse (from low-risk to high-risk lifestyle), and better (from high-risk to low-risk lifestyle).
*The concerned diseases include cardiovascular diseases, cancer, and chronic respiratory diseases (including chronic obstructive pulmonary disease and asthma).
†Difference in physical activity between baseline and resurvey.

Supplementary Table 3. Sensitivity analysis of associations of the number of low-risk lifestyle factors with each transition in men and women separately.

	HRs (95% CIs)					
	0-1	2	3	4	5	Per 1-factor increase
**Men**						
**Baseline → disease**						
Different interval						
0.5 days	1.00 (Referent)	0.80 (0.78-0.81)	0.69 (0.67-0.71)	0.61 (0.59-0.63)	0.56 (0.50-0.63)	0.85 (0.84-0.86)
0.5 years	1.00 (Referent)	0.80 (0.78-0.81)	0.69 (0.67-0.71)	0.61 (0.59-0.63)	0.57 (0.51-0.63)	0.85 (0.84-0.86)
1 year	1.00 (Referent)	0.80 (0.78-0.81)	0.69 (0.67-0.71)	0.61 (0.59-0.63)	0.57 (0.51-0.63)	0.85 (0.84-0.86)
Regarding the participants who died on the same date of disease onset as death without disease onset	1.00 (Referent)	0.80 (0.78-0.81)	0.70 (0.68-0.72)	0.63 (0.60-0.65)	0.57 (0.51-0.64)	0.85 (0.85-0.86)
Excluding participants who died on the same date of disease onset from the analysis	1.00 (Referent)	0.79 (0.77-0.81)	0.69 (0.67-0.71)	0.62 (0.60-0.64)	0.57 (0.51-0.64)	0.85 (0.84-0.86)
Excluding events occurred in the first two-year of follow-up	1.00 (Referent)	0.80 (0.78-0.82)	0.70 (0.68-0.72)	0.62 (0.60-0.64)	0.56 (0.49-0.63)	0.85 (0.84-0.86)
Additional adjustment for hypertension, diabetes, and usage of antihypertensive and glucose-lowering medications and statin	1.00 (Referent)	0.84 (0.82-0.85)	0.74 (0.72-0.76)	0.66 (0.64-0.68)	0.61 (0.55-0.69)	0.87 (0.86-0.88)
**Baseline → death**[*]						
Different interval						
0.5 days	1.00 (Referent)	0.80 (0.72-0.89)	0.73 (0.65-0.81)	0.64 (0.55-0.74)	0.40 (0.20-0.80)	0.86 (0.83-0.90)
0.5 years	1.00 (Referent)	0.80 (0.72-0.89)	0.73 (0.65-0.81)	0.64 (0.55-0.74)	0.40 (0.20-0.80)	0.86 (0.83-0.90)
1 year	1.00 (Referent)	0.80 (0.72-0.88)	0.72 (0.65-0.81)	0.64 (0.55-0.74)	0.40 (0.20-0.80)	0.86 (0.82-0.90)
Regarding the participants who died on the same date of disease onset as death without disease onset	1.00 (Referent)	0.79 (0.75-0.84)	0.65 (0.61-0.69)	0.53 (0.48-0.58)	0.42 (0.28-0.63)	0.81 (0.79-0.83)
Excluding participants who died on the same date of disease onset from the analysis	1.00 (Referent)	0.79 (0.71-0.88)	0.72 (0.64-0.80)	0.62 (0.54-0.73)	0.39 (0.19-0.79)	0.86 (0.82-0.89)

	HRs (95% CIs)					
	0-1	2	3	4	5	Per 1-factor increase
Excluding events occurred in the first two-year of follow-up	1.00 (Referent)	0.82 (0.73-0.92)	0.75 (0.66-0.84)	0.68 (0.58-0.81)	0.47 (0.23-0.95)	0.88 (0.84-0.92)
Additional adjustment for hypertension, diabetes, and usage of antihypertensive and glucose-lowering medications and statin	1.00 (Referent)	0.83 (0.75-0.93)	0.77 (0.69-0.86)	0.68 (0.58-0.79)	0.43 (0.22-0.87)	0.88 (0.84-0.92)
**Disease → death**[†]						
Different interval						
0.5 days	1.00 (Referent)	0.92 (0.88-0.96)	0.83 (0.79-0.86)	0.74 (0.69-0.78)	0.64 (0.50-0.83)	0.90 (0.89-0.92)
0.5 years	1.00 (Referent)	0.92 (0.88-0.96)	0.83 (0.79-0.86)	0.73 (0.69-0.78)	0.65 (0.51-0.84)	0.90 (0.89-0.92)
1 year	1.00 (Referent)	0.92 (0.88-0.96)	0.82 (0.79-0.86)	0.74 (0.69-0.78)	0.66 (0.51-0.84)	0.90 (0.89-0.92)
Regarding the participants who died on the same date of disease onset as death without disease onset	1.00 (Referent)	0.90 (0.86-0.94)	0.80 (0.76-0.85)	0.72 (0.67-0.78)	0.62 (0.46-0.82)	0.90 (0.88-0.92)
Excluding participants who died on the same date of disease onset from the analysis	1.00 (Referent)	0.90 (0.86-0.94)	0.80 (0.76-0.85)	0.72 (0.67-0.78)	0.62 (0.46-0.82)	0.90 (0.88-0.92)
Excluding events occurred in the first two-year of follow-up	1.00 (Referent)	0.91 (0.87-0.95)	0.82 (0.78-0.86)	0.74 (0.69-0.80)	0.66 (0.50-0.87)	0.90 (0.89-0.92)
Additional adjustment for hypertension, diabetes, and usage of antihypertensive and glucose-lowering medications and statin	1.00 (Referent)	0.92 (0.88-0.96)	0.83 (0.79-0.87)	0.74 (0.69-0.79)	0.65 (0.50-0.83)	0.91 (0.89-0.92)
**Women**						
**Baseline → disease**						
Different interval						
0.5 days	1.00 (Referent)	0.93 (0.87-0.99)	0.78 (0.73-0.83)	0.69 (0.65-0.74)	0.69 (0.64-0.75)	0.88 (0.87-0.89)
0.5 years	1.00 (Referent)	0.93 (0.87-0.99)	0.78 (0.73-0.83)	0.70 (0.65-0.74)	0.69 (0.64-0.75)	0.88 (0.87-0.89)
1 year	1.00 (Referent)	0.93 (0.87-0.99)	0.78 (0.73-0.83)	0.69 (0.65-0.74)	0.69 (0.64-0.75)	0.88 (0.87-0.89)
Regarding the participants who died on the same date of disease onset as death without	1.00 (Referent)	0.94 (0.88-1.01)	0.80 (0.75-0.85)	0.72 (0.67-0.77)	0.71 (0.66-0.77)	0.88 (0.88-0.89)

	HRs (95% CIs)					
	0-1	2	3	4	5	Per 1-factor increase
disease onset						
Excluding participants who died on the same date of disease onset from the analysis	1.00 (Referent)	0.93 (0.87-1.00)	0.78 (0.73-0.84)	0.71 (0.66-0.75)	0.70 (0.65-0.76)	0.88 (0.87-0.89)
Excluding events occurred in the first two-year of follow-up	1.00 (Referent)	0.93 (0.87-0.99)	0.78 (0.73-0.84)	0.71 (0.66-0.76)	0.71 (0.65-0.77)	0.89 (0.88-0.90)
Additional adjustment for hypertension, diabetes, and usage of antihypertensive and glucose-lowering medications and statin	1.00 (Referent)	0.91 (0.85-0.96)	0.81 (0.76-0.86)	0.74 (0.70-0.79)	0.74 (0.68-0.80)	0.91 (0.90-0.92)
**Baseline → death**[*]						
Different interval						
0.5 days	1.00 (Referent)	0.97 (0.68-1.40)	0.81 (0.57-1.16)	0.65 (0.45-0.94)	0.57 (0.34-0.94)	0.83 (0.78-0.88)
0.5 years	1.00 (Referent)	0.97 (0.67-1.40)	0.81 (0.56-1.16)	0.65 (0.45-0.93)	0.57 (0.34-0.94)	0.83 (0.78-0.88)
1 year	1.00 (Referent)	0.97 (0.67-1.40)	0.81 (0.56-1.16)	0.65 (0.45-0.93)	0.57 (0.34-0.94)	0.83 (0.78-0.88)
Regarding the participants who died on the same date of disease onset as death without disease onset	1.00 (Referent)	0.80 (0.66-0.96)	0.63 (0.52-0.76)	0.50 (0.41-0.60)	0.44 (0.32-0.59)	0.79 (0.77-0.82)
Excluding participants who died on the same date of disease onset from the analysis	1.00 (Referent)	0.96 (0.67-1.39)	0.80 (0.56-1.15)	0.64 (0.44-0.92)	0.56 (0.34-0.92)	0.82 (0.78-0.87)
Excluding events occurred in the first two-year of follow-up	1.00 (Referent)	1.01 (0.67-1.53)	0.86 (0.57-1.29)	0.70 (0.46-1.05)	0.68 (0.40-1.17)	0.84 (0.79-0.90)
Additional adjustment for hypertension, diabetes, and usage of antihypertensive and glucose-lowering medications and statin	1.00 (Referent)	0.95 (0.66-1.38)	0.85 (0.59-1.22)	0.71 (0.49-1.02)	0.62 (0.37-1.03)	0.87 (0.82-0.92)
**Disease → death**[†]						
Different interval						
0.5 days	1.00 (Referent)	0.78 (0.69-0.88)	0.74 (0.66-0.83)	0.69 (0.62-0.78)	0.57 (0.47-0.69)	0.93 (0.91-0.95)
0.5 years	1.00 (Referent)	0.78 (0.69-0.88)	0.74 (0.66-0.84)	0.70 (0.62-0.79)	0.57 (0.48-0.69)	0.93 (0.91-0.95)
1 year	1.00 (Referent)	0.78 (0.69-0.88)	0.74 (0.66-0.83)	0.71 (0.63-0.80)	0.57 (0.48-0.69)	0.93 (0.91-0.95)

	HRs (95% CIs)					
	0-1	2	3	4	5	Per 1-factor increase
Regarding the participants who died on the same date of disease onset as death without disease onset	1.00 (Referent)	0.76 (0.66-0.88)	0.74 (0.64-0.84)	0.71 (0.62-0.82)	0.57 (0.46-0.71)	0.94 (0.92-0.97)
Excluding participants who died on the same date of disease onset from the analysis	1.00 (Referent)	0.76 (0.66-0.88)	0.74 (0.64-0.84)	0.71 (0.62-0.82)	0.57 (0.46-0.71)	0.94 (0.92-0.97)
Excluding events occurred in the first two-year of follow-up	1.00 (Referent)	0.77 (0.68-0.88)	0.74 (0.65-0.85)	0.71 (0.62-0.81)	0.58 (0.47-0.71)	0.94 (0.91-0.96)
Additional adjustment for hypertension, diabetes, and usage of antihypertensive and glucose-lowering medications and statin	1.00 (Referent)	0.78 (0.70-0.88)	0.76 (0.67-0.85)	0.72 (0.64-0.81)	0.59 (0.49-0.71)	0.94 (0.92-0.96)

HR indicates hazard ratio; CI, confidence interval.

Cox regression was used to estimate the HRs and 95% CIs, with adjustment for education, marital status, family histories of heart attack, stroke, and cancer, and menopausal status (women only) as appropriate. In the analysis of transitions from a disease state to all-cause mortality, HRs were further adjusted for age at diagnosis of corresponding diseases (years). All statistical tests were two-sided.

The definition of low-risk lifestyle factors was the same as in Supplementary Table 1. The disease event in this analysis refers to the first occurrence of any of cardiovascular diseases, cancer, and chronic respiratory diseases (including chronic obstructive pulmonary disease and asthma).

[*]The transition from baseline to death refers to the transition from a disease-free state at baseline to death from any cause other than the concerned disease without experiencing disease onset.

[†]The transition from disease to death refers to the transition from the concerned disease state to all-cause death.

Supplementary Table 4. Life expectancy at ages 40, 50, and 65 years without chronic diseases and life expectancy differences by the number of low-risk lifestyle factors in men and women separately.

	Men		Women	
	LE without diseases, 95% CIs	Difference, 95% CIs	LE without diseases, 95% CIs	Difference, 95% CIs
**At age 40 years**				
0-1	23.9 (23.2, 24.6)	Referent	24.2 (23.5, 24.9)	Referent
2	26.3 (25.6, 27.1)	2.4 (2.2, 2.7)	25.0 (24.1, 26.0)	0.8 (0.1, 1.5)
3	27.9 (27.1, 28.6)	4.0 (3.7, 4.2)	27.0 (26.2, 27.8)	2.8 (2.1, 3.5)
4	29.2 (28.4, 30.0)	5.3 (4.9, 5.6)	28.3 (27.4, 29.2)	4.1 (3.4, 4.8)
5	30.2 (28.8, 31.6)	6.3 (5.1, 7.5)	28.4 (27.2, 29.6)	4.2 (3.3, 5.1)
**At age 50 years**				
0-1	17.1 (16.5, 17.7)	Referent	17.6 (17.0, 18.2)	Referent
2	19.1 (18.5, 19.8)	2.1 (1.9, 2.3)	18.3 (17.5, 19.1)	0.7 (0.1, 1.3)
3	20.4 (19.8, 21.1)	3.4 (3.2, 3.6)	20.0 (19.3, 20.7)	2.4 (1.8, 3.0)
4	21.6 (20.9, 22.3)	4.5 (4.2, 4.8)	21.1 (20.3, 21.9)	3.5 (2.9, 4.1)
5	22.5 (21.2, 23.7)	5.4 (4.4, 6.5)	21.2 (20.2, 22.2)	3.6 (2.8, 4.4)
**At age 65 years**				
0-1	9.1 (8.7, 9.6)	Referent	9.9 (9.4, 10.3)	Referent
2	10.5 (10.0, 11.0)	1.4 (1.3, 1.6)	10.4 (9.8, 10.9)	0.5 (0.1, 0.9)
3	11.4 (10.9, 11.9)	2.3 (2.2, 2.5)	11.5 (11.0, 12.1)	1.6 (1.2, 2.0)
4	12.2 (11.7, 12.8)	3.1 (2.9, 3.3)	12.3 (11.7, 12.9)	2.4 (2.0, 2.9)
5	12.9 (12.0, 13.8)	3.7 (3.0, 4.5)	12.4 (11.6, 13.1)	2.5 (1.9, 3.0)

LE indicates life expectancy; CI, confidence interval.

Chronic diseases in this analysis refer to a combination of cardiovascular diseases, cancer, and chronic respiratory diseases, of which only the first occurrence was considered.

The definition of low-risk lifestyle factors was the same as in Supplementary Table 1.

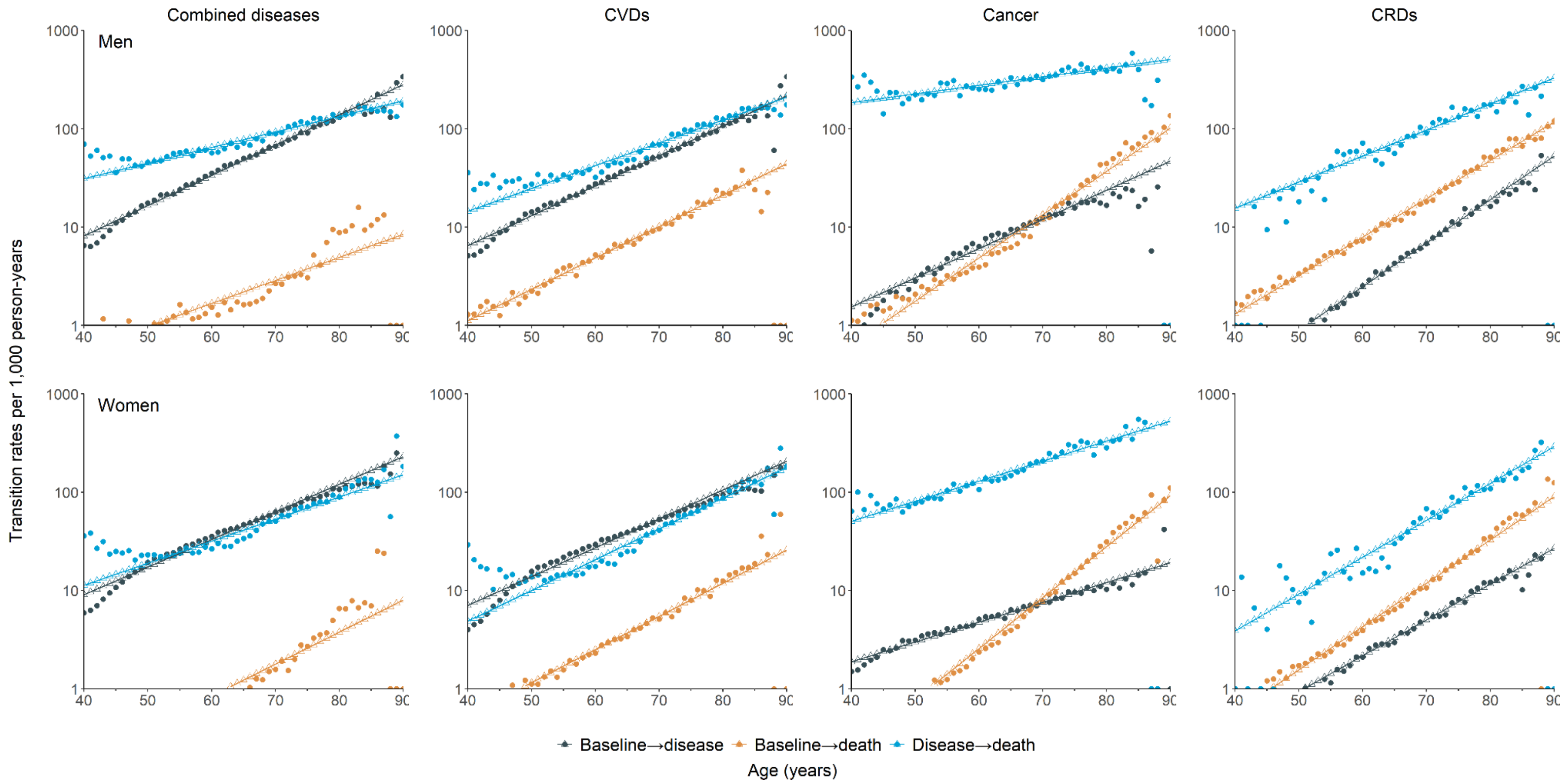

Supplementary Figure 1. Observed and predicted transition rates of CKB participants in men (n=181,544) and women (n=269,689) separately.

CVDs indicate cardiovascular diseases; CRDs, chronic respiratory diseases, including chronic obstructive pulmonary disease and asthma. Combined diseases include CVDs, cancer, and CRDs. The hollow triangle represents predicted values and the solid circle represents observed values.

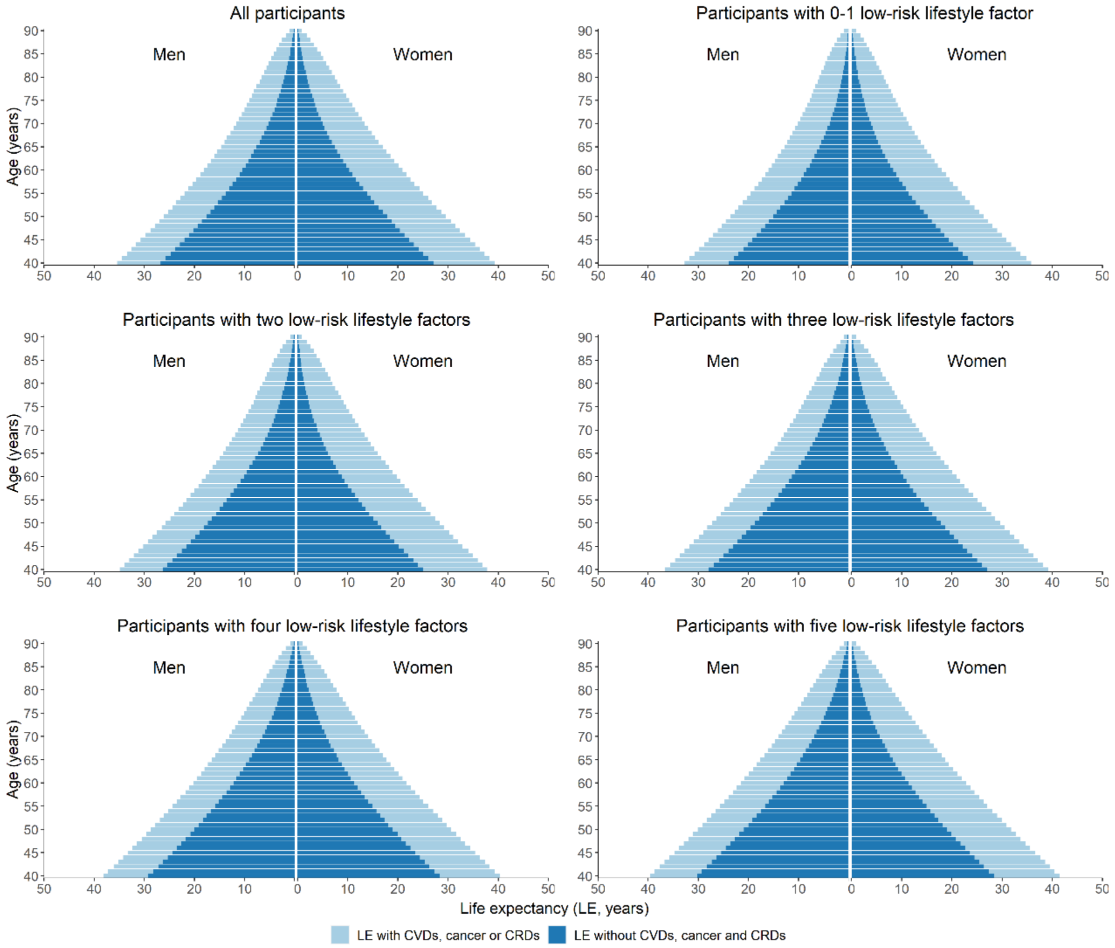

Supplementary Figure 2. Life expectancy at every age after age 40 years with and without cardiovascular diseases (CVDs), cancer, and/or chronic respiratory diseases (CRDs), conditional on disease-free at age 40 years and by the number of low-risk lifestyle factors in men (n=181,544) and women (n=269,689) separately. The definition of low-risk lifestyle factors was the same as in Supplementary Table 1.

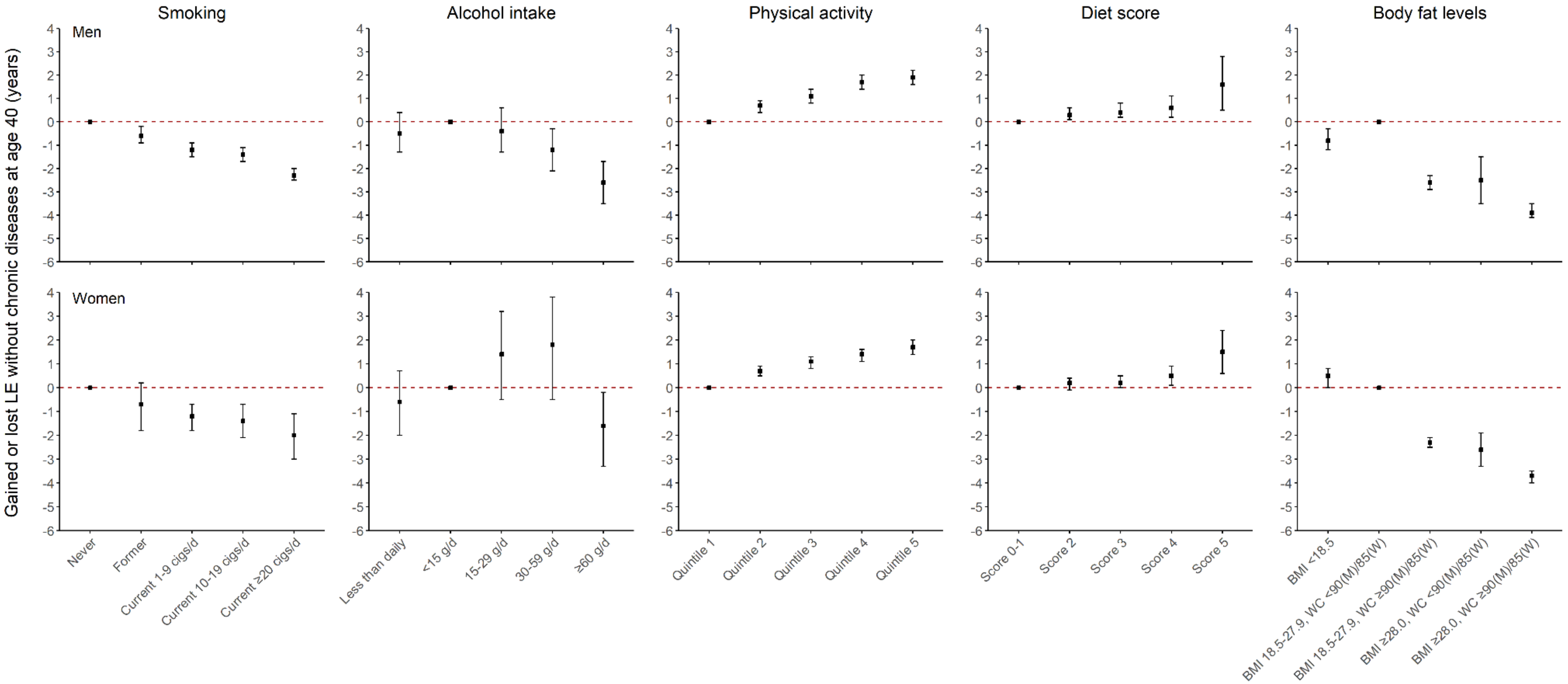

Supplementary Figure 3. Projected gained or lost life expectancy (LE) at age 40 years without cardiovascular diseases (CVDs), cancer, and chronic respiratory diseases (CRDs) by levels of individual lifestyle risk factors in men (n=181,544) and women (n=269,689) separately.

Cigs indicate cigarettes or equivalent; BMI, body mass index; WC, waist circumference; M, men; W, women.

Former smokers refer to those having stopped smoking for reasons other than illness. Participants who had stopped smoking due to illness were classified as current smokers. Less than daily group included never-regular drinkers and current weekly drinkers. Former alcohol drinkers were included in the heavy drinking category (≥60 g of pure alcohol per day). Physical activity level was categorized based on age- (<50 years, 50-59 years, and ≥60 years) and sex-specific quintile of total physical activity level. Diet score was created

based on the following criteria: eating fresh vegetables daily, eating fresh fruits daily, eating red meat 1-6 days per week, eating legumes ≥4 days per week, and eating fish ≥1 day per week. For each food group, the participant who met the criterion received a score of 1, and otherwise, 0. The error bars represent 95% CIs.

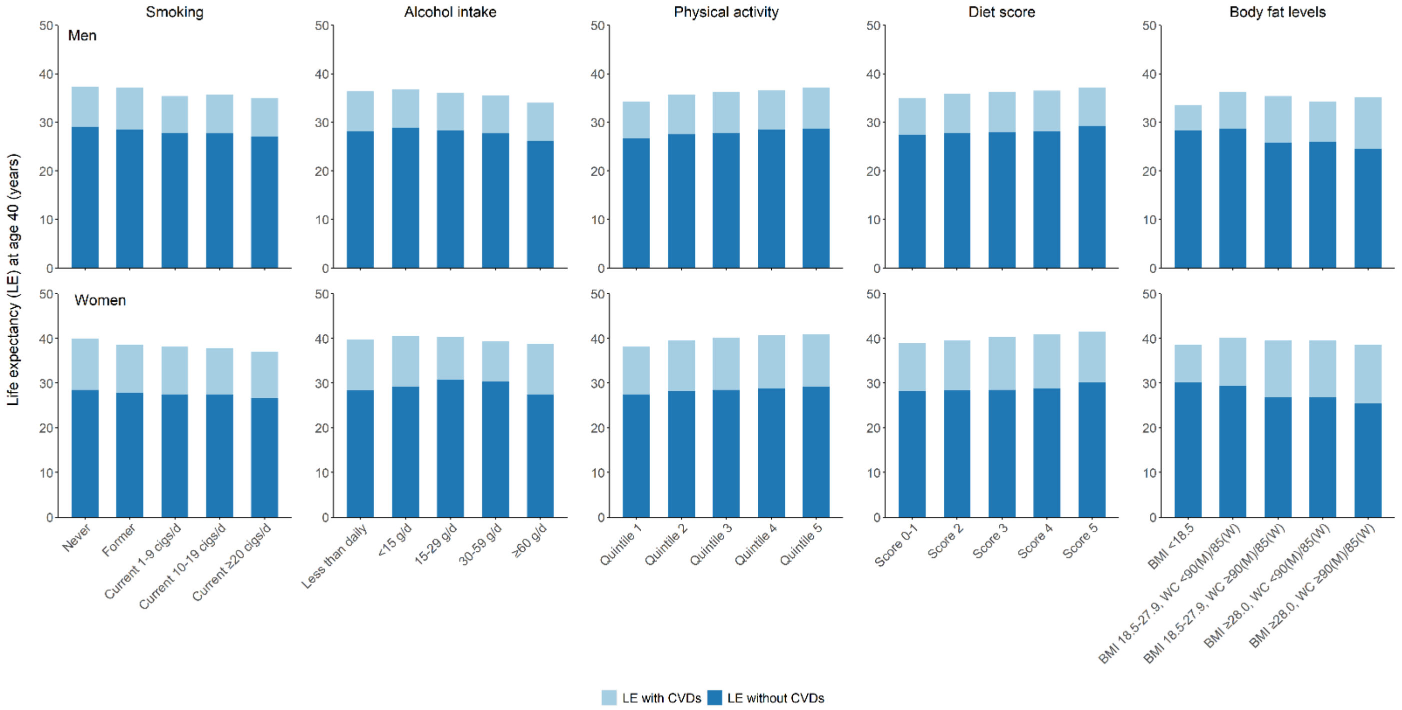

LE without CVDs / total LE (%)

	Never	Former	Current 1-9 cigs/d	Current 10-19 cigs/d	Current ≥20 cigs/d	Less than daily	<15 g/d	15-29 g/d	30-59 g/d	≥60 g/d	Quintile 1	Quintile 2	Quintile 3	Quintile 4	Quintile 5	Score 0-1	Score 2	Score 3	Score 4	Score 5	BMI <18.5	BMI 18.5-27.9, WC <90(M)/85(W)	BMI 18.5-27.9, WC ≥90(M)/85(W)	BMI ≥28.0, WC <90(M)/85(W)	BMI ≥28.0, WC ≥90(M)/85(W)
Men	77.7	76.8	78.5	77.6	77.4	77.5	78.3	78.6	78.0	77.1	77.8	77.0	76.8	77.9	77.2	78.3	77.4	77.1	77.0	78.7	84.2	79.0	72.9	75.5	69.8
Women	71.4	72.3	72.0	72.8	72.2	71.4	71.9	76.2	77.1	70.6	71.7	71.4	70.9	70.8	71.2	72.3	71.7	70.7	70.2	72.5	78.2	72.9	68.1	67.8	65.8

Supplementary Figure 4. Life expectancy at age 40 years with and without cardiovascular diseases (CVDs) by levels of individual lifestyle risk factors in men (n=181,544) and women (n=269,689) separately.

Cigs indicate cigarettes or equivalent; BMI, body mass index; WC, waist circumference; M, men; W, women. The definition for categories of each lifestyle factor was the same as in Supplementary Figure 3.

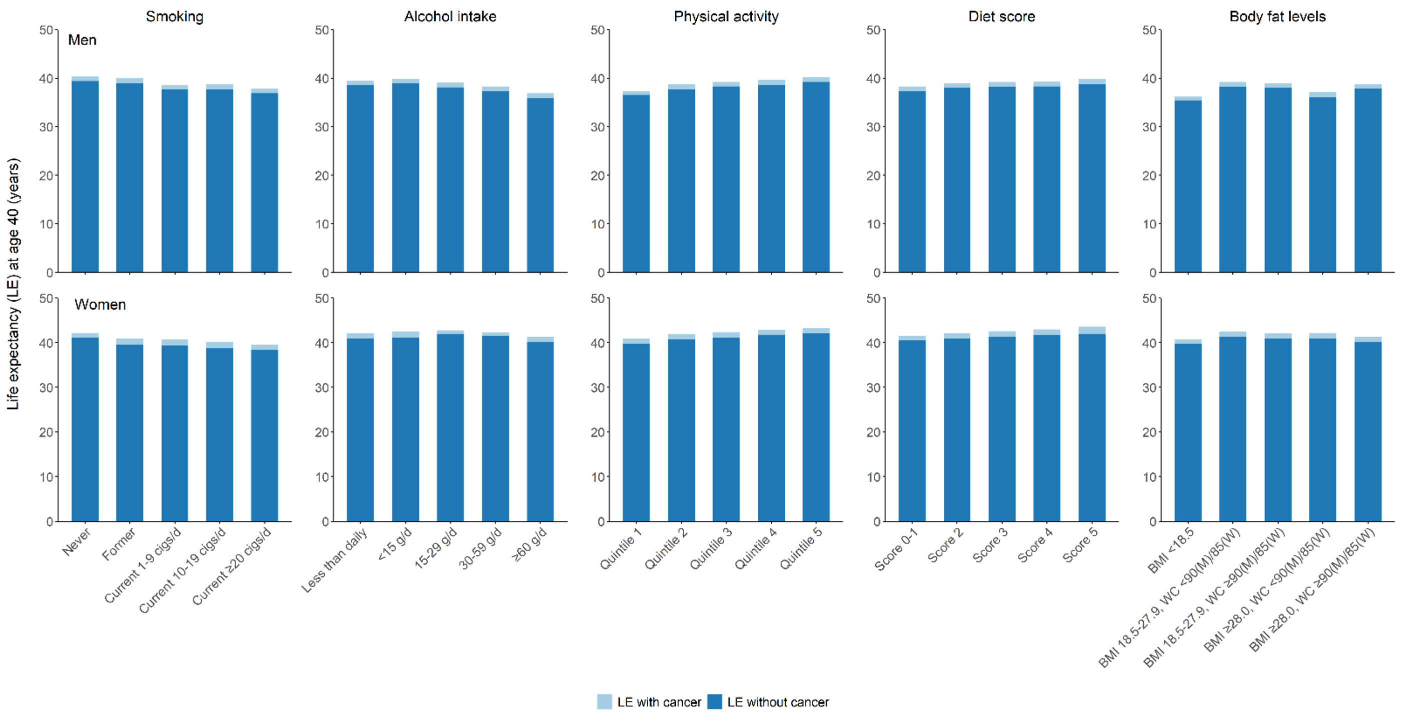

LE without cancer / total LE (%)

Men	97.8	97.5	97.7	97.4	97.4	97.7	97.5	97.4	97.4	97.3	97.6	97.4	97.7	97.5	97.5	97.7	97.7	97.4	97.5	97.2	97.5	97.4	97.4	97.0	97.7
Women	97.4	96.6	96.8	96.8	97.0	97.4	96.7	98.1	98.1	97.1	97.3	97.1	97.2	97.4	97.2	97.6	97.4	97.2	97.0	96.3	97.5	97.4	97.1	96.9	96.9

Supplementary Figure 5. Life expectancy at age 40 years with and without cancer by levels of individual lifestyle risk factors in men (n=181,544) and women (n=269,689) separately.
Cigs indicate cigarettes or equivalent; BMI, body mass index; WC, waist circumference; M, men; W, women. The definition for categories of each lifestyle factor was the same as in Supplementary Figure 3.

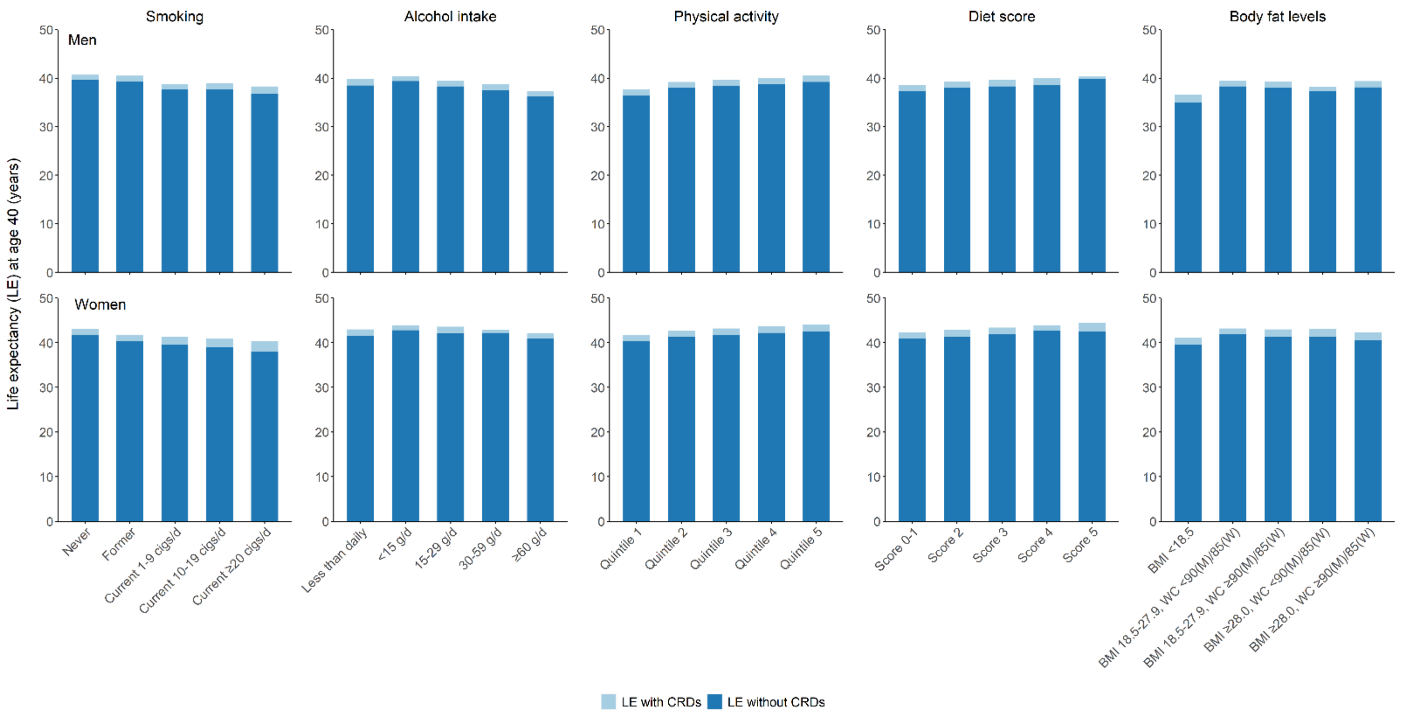

LE without CRDs / total LE (%)

Men	97.5	97.0	97.2	96.4	96.1	96.7	97.5	97.0	96.6	97.1	96.6	96.9	96.7	97.0	96.6	96.9	96.7	96.7	96.5	99.0	95.4	97.0	96.7	97.6	96.7
Women	96.8	96.4	95.7	95.1	94.3	96.5	97.7	96.6	98.4	97.4	96.6	97.0	96.5	96.6	96.4	96.7	96.5	96.5	97.3	95.7	95.9	97.0	96.3	95.8	96.0

Supplementary Figure 6. Life expectancy at age 40 years with and without chronic respiratory diseases (CRDs) by levels of individual lifestyle risk factors in men (n=181,544) and women (n=269,689) separately.

Cigs indicate cigarettes or equivalent; BMI, body mass index; WC, waist circumference; M, men; W, women. The definition for categories of each lifestyle factor was the same as in Supplementary Figure 3.

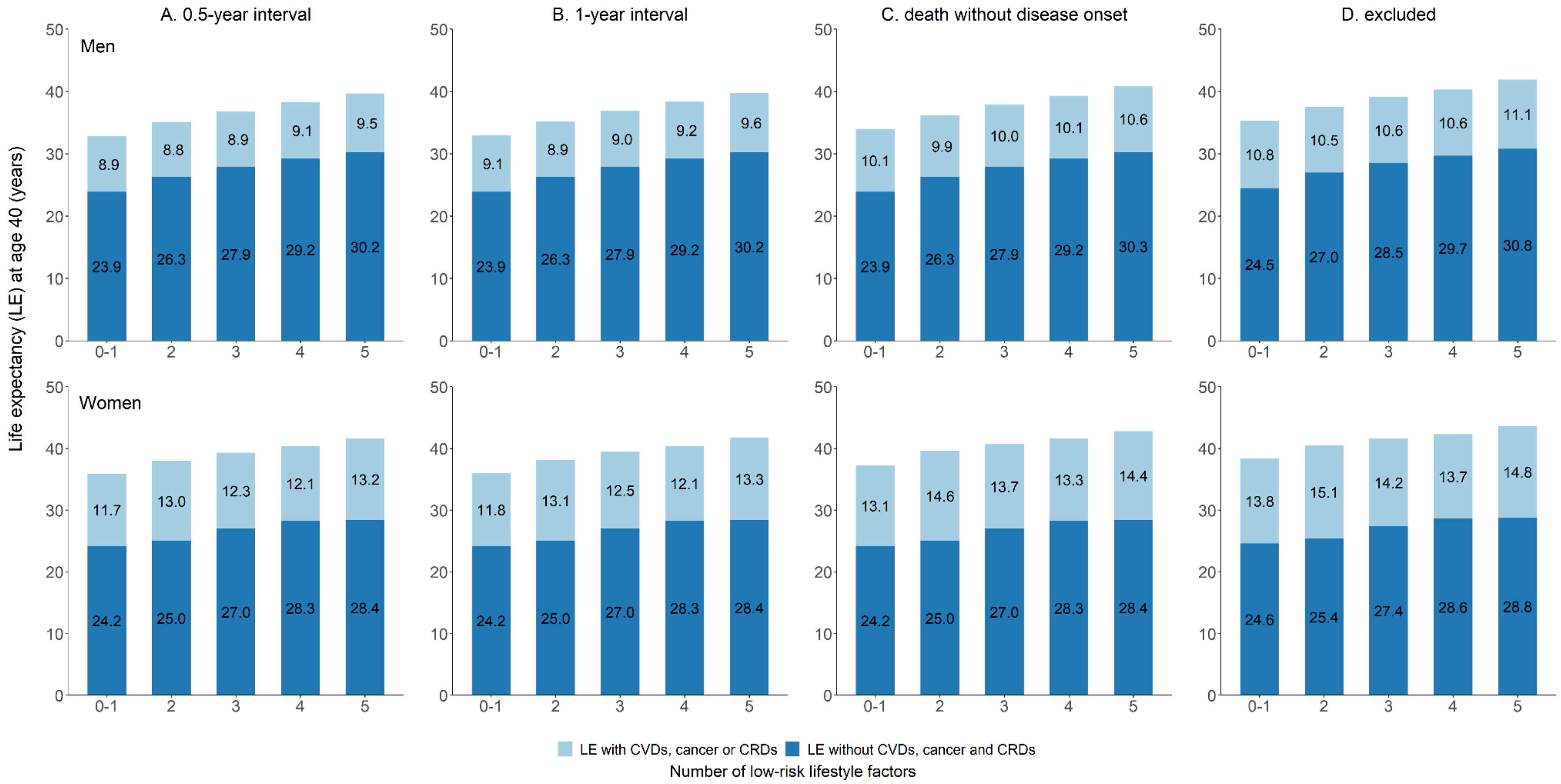

Supplementary Figure 7. Sensitivity analysis of life expectancy at age 40 years with and without cardiovascular diseases (CVDs), cancer, and/or chronic respiratory diseases (CRDs) by the number of low-risk lifestyle factors.
(A) by assigning the date of disease onset as the date of death minus 0.5 years for participants who died on the same date of disease onset (n=451,233); (B) by assigning the date of disease onset as the date of death minus 1 year for participants who died on the same date of disease onset (n=451,233); (C) by regarding the participants who died on the same date of disease onset as death without disease onset (n=451,233); (D) by excluding participants who died on the same date of disease onset from the analysis (n=442,030).

The definition of low-risk lifestyle factors was the same as in Supplementary Table 1.

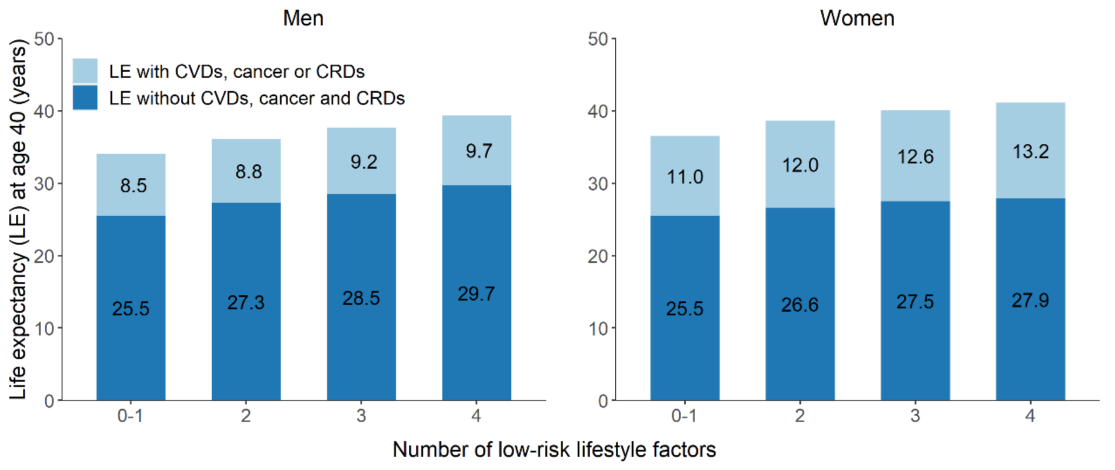

LE without CVDs, cancer and CRDs / total LE (%)

Men	75.0	75.6	75.6	75.4	*Women*	69.9	68.9	68.6	67.9

Supplementary Figure 8. Sensitivity analysis of life expectancy at age 40 years with and without cardiovascular diseases (CVDs), cancer, and/or chronic respiratory diseases (CRDs) by the number of low-risk lifestyle factors without considering body fat levels in men (n=181,544) and women (n=269,689) separately. The definition of low-risk lifestyle factors was the same as in Supplementary Table 1 without considering body fat levels.

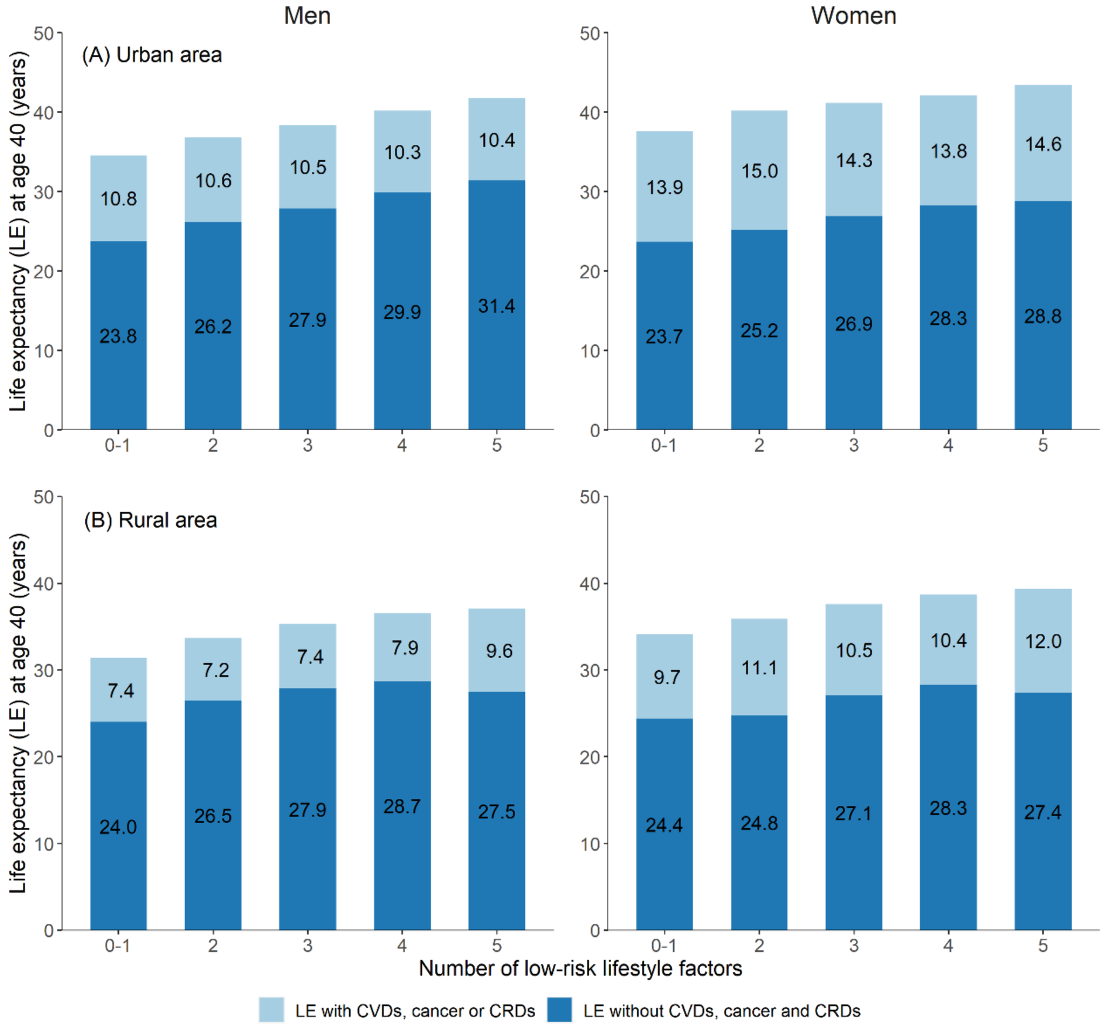

LE without CVDs, cancer, and CRDs / total LE (%)

Urban	68.8	71.2	72.7	74.4	75.1	63.0	62.7	65.3	67.2	66.4
Rural	76.4	78.6	79.0	78.4	74.1	71.6	69.1	72.1	73.1	69.5

Supplementary Figure 9. Life expectancy at age 40 years with and without cardiovascular diseases (CVDs), cancer, and/or chronic respiratory diseases (CRDs) by the number of low-risk lifestyle factors stratified by residence.
(A) urban area (n=196,725); (B) rural area (n=254,508).
The definition of low-risk lifestyle factors was the same as in Supplementary Table 1.

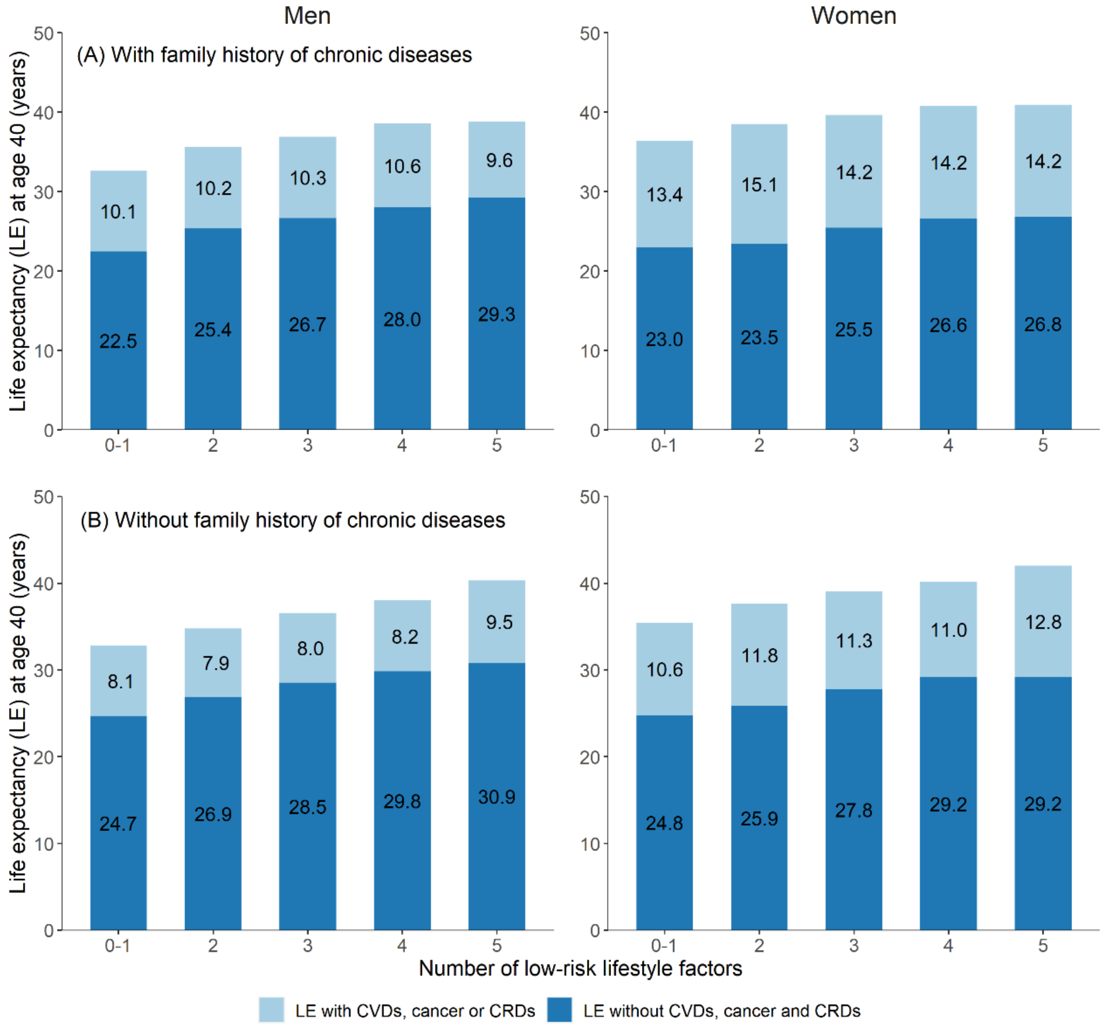

LE without CVDs, cancer, and CRDs / total LE (%)

With	69.0	71.2	72.2	72.5	75.4	63.2	60.9	64.3	65.2	65.4
Without	75.3	77.3	78.0	78.5	76.5	70.0	68.7	71.1	72.7	69.5

Supplementary Figure 10. Life expectancy at age 40 years with and without cardiovascular diseases (CVDs), cancer, and/or chronic respiratory diseases (CRDs) by the number of low-risk lifestyle factors stratified by family history of chronic diseases.

(A) with a family history of chronic diseases (n=150,465); (B) without family history of chronic diseases (n=300,768).

A family history of chronic diseases was considered present if the participant had a family history of either heart attack, stroke, or cancer. The definition of low-risk lifestyle factors was the same as in Supplementary Table 1.

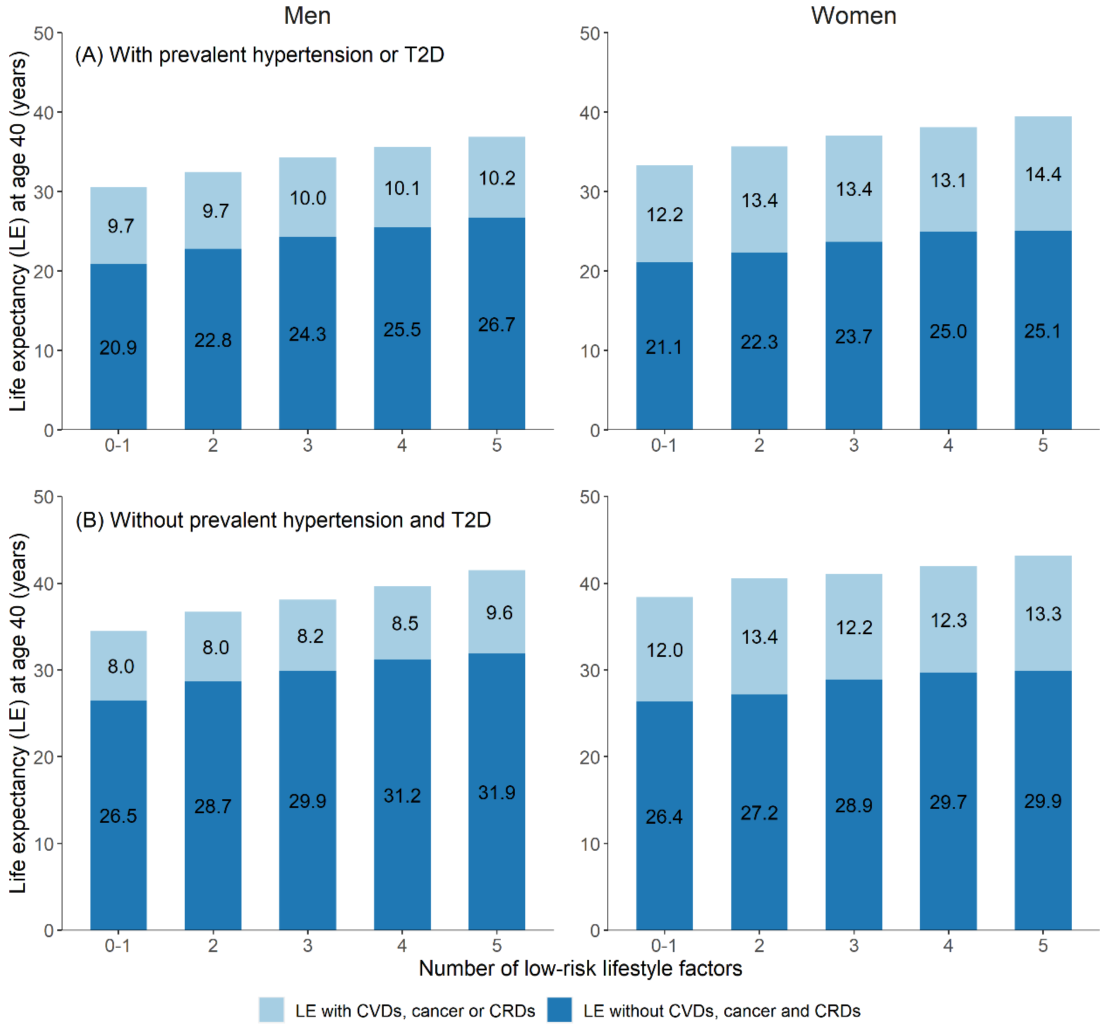

LE without CVDs, cancer, and CRDs / total LE (%)

With	68.3	70.2	70.8	71.6	72.4	63.4	62.5	63.9	65.6	63.5
Without	76.8	78.2	78.5	78.6	76.9	68.8	67.0	70.3	70.7	69.2

Supplementary Figure 11. Life expectancy at age 40 years with and without cardiovascular diseases (CVDs), cancer, and/or chronic respiratory diseases (CRDs) by the number of low-risk lifestyle factors stratified by prevalent hypertension and/or type 2 diabetes (T2D) at baseline.

(A) with prevalent hypertension or T2D (n=160,369); (B) without prevalent hypertension and T2D (n=290,864).

The definition of low-risk lifestyle factors was the same as in Supplementary Table 1.

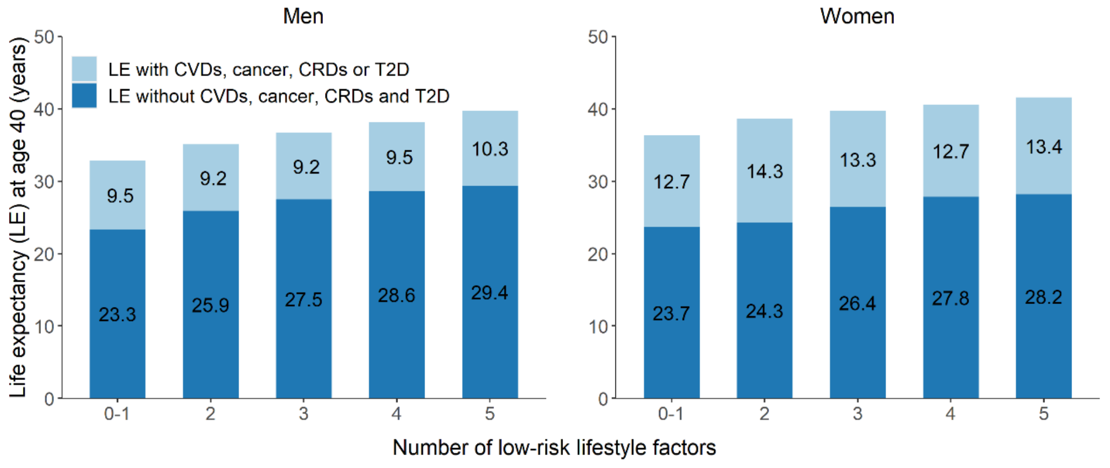

LE without CVDs, cancer, CRDs and T2D / total LE (%)

Men	71.0	73.9	74.9	75.0	74.0	*Women*	65.1	62.9	66.6	68.7	67.8

Supplementary Figure 12. Life expectancy at age 40 years with and without cardiovascular diseases (CVDs), cancer, chronic respiratory diseases (CRDs), and/or type 2 diabetes (T2D) by the number of low-risk lifestyle factors in men (n=172,292) and women (n=254,807) separately.

The definition of low-risk lifestyle factors was the same as in Supplementary Table 1. In this analysis, participants with T2D at baseline were excluded (n=24,134).
